# Intravenous thrombolysis in acute ischemic stroke patients with pre‐stroke disability: A systematic review and meta‐analysis

**DOI:** 10.1002/brb3.3431

**Published:** 2024-02-15

**Authors:** Qiangji Bao, Xinting Wu, Yiming Li, Shujun Chen, Qiang Zhang, Mingfei Yang, Peng Yang

**Affiliations:** ^1^ Department of Neurosurgery Guang'an People's Hospital Guang'an Sichuan China; ^2^ Department of Anesthesia Guang'an People's Hospital Guang'an Sichuan China; ^3^ Graduate School Qinghai University Xining Qinghai China; ^4^ Department of Neurosurgery Qinghai Provincial People's Hospital Xining Qinghai China

**Keywords:** intravenous thrombolysis, ischemic stroke, meta‐analysis, outcome, pre‐stroke disability

## Abstract

**Background:**

Intravenous thrombolysis (IVT), which is safe and effective, is the first‐line therapy for acute ischemic stroke (AIS). However, its benefit for AIS patients with pre‐stroke disability (PSD) is controversial.

**Objective:**

We determined the association of PSD with the safety and efficacy of IVT among patients with AIS.

**Methods:**

We searched PubMed, Embase, and the Cochrane Library from inception to May 23, 2022. The articles focusing on outcomes of AIS patients with PSD receiving IVT were retrieved. We used the random‐effects model to pool outcomes including mortality, 24 h NIHSS improvement, symptomatic intracerebral hemorrhage (sICH), favorable functional outcome (FFO), the favorable outcome, and mortality prevalence.

**Results:**

Ten studies (including 245,773 participants) that reported the outcomes of AIS patients with PSD undergoing IVT were included. In unadjusted analyses, PSD was associated with mortality (10 studies; odds ratio [OR] 1.739, 95% confidence interval [CI], 1.336–2.407), FFO (7 studies; OR 1.057, 95% CI, 1.015–1.100), 24 h NIHSS improvement (5 studies; OR .840, 95% CI, .819–.917, *p* = .000), and sICH (9 studies; OR .773, 95% CI, .481–1.243). In adjusted analyses, PSD was associated with mortality (seven studies; OR_adj_ 1.789, 95% CI, 1.413–2.264), FFO (five studies; OR_adj_ 1.087, 95% CI, 1.002–1.179), 24 h NIHSS improvement (five studies; OR_adj_ .837, 95% CI, .799–.876), and sICH (five studies; OR_adj_ .857, 95% CI, .725–1.012). The prevalence of FFO and mortality in patients with pre‐stroke modified Rankin Scale scores of 2–5 were 49% (0.42–0.56) and 37% (0.21–0.53), respectively.

**Conclusions:**

Patients with PSD undergoing IVT had a higher mortality rate than those without PSD. Meanwhile, PSD was associated with FFO, and there was no significant difference in sICH and 24 h NIHSS improvement. High‐quality data are needed to clarify the benefits of administering IVT in these patients.

## Graphical abstract

A total of 10 studies with more than 245,773 subjects were included in this systematic review and meta‐analysis, which was the largest study published to date probing the clinical significance of intravenous thrombolysis in acute ischemic stroke patients with pre‐stroke disability.

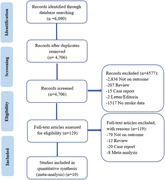

## 1 INTRODUCTION

Stroke is still the leading cause of neurological disability in almost all regions, including Europe (The Lancet Neurology, 2020). Since 2015, stroke has become the leading cause of death and disability in China, posing a significant threat to the health of its citizens as a major chronic noncommunicable disease (Tu & Wang, 2023; Tu et al., 2023). The pathogenesis of acute ischemic stroke (AIS) is considered to be the thrombotic occlusion of the cerebral artery. Intravenous thrombolysis (IVT) with recombinant tissue plasminogen activator, a routine treatment for patients with AIS, can reduce disability and significantly increase the overall likelihood of a good stroke outcome at 3–6 months, and there are increasing proportional benefits with earlier treatment (Emberson et al., 2014; Wardlaw et al., 2009). The current guidelines (Berge et al., 2021) suggest that IVT remains the cornerstone of AIS treatment. However, systemic thrombolysis in the early phase can cause complications. The most significant complication is symptomatic intracranial hemorrhage (sICH), and various confounding factors, such as age, history of premorbid disability, and diabetes mellitus, can increase the risk of sICH (Lansberg et al., 2007). The application of IVT is still limited in patients with pre‐stroke disability (PSD) for several reasons, including a large number of contraindications. Although some studies have recommended IVT for eligible patients with AIS and a history of PSD (Hacke et al., 2008; National Institute of Neurological Disorders & Stroke rt‐PA Stroke Study Group, 1995), clinicians remain uncertain about the risks related to emergent decisions with IVT administration, especially in patients with moderate‐to‐severe PSD. Therefore, there are insufficient clinical data to routinely recommend IVT treatment for AIS with PSD.

We, therefore, performed a systematic review and meta‐analysis to further comparatively assess the safety and effectiveness of IVT for AIS patients with PSD.

## 2 METHODS

Our meta‐analysis was conducted according to the Preferred Reporting Items for Systematic Reviews and Meta‐Analyses statement (Moher et al., 2009) and was reported following the Meta‐Analysis of Observational Studies in Epidemiology (Stroup et al., 2000) (Supporting Information 1). The review protocol was prospectively registered in the International Platform of Registered Systematic Review and Meta‐analysis Protocols (INPLASY, https://inplasy.com/) under the number INPLASY202280098.

### 2.1 Data sources and database searches

We performed a systematic search to screen studies focusing on the outcomes of AIS patients with PSD receiving IVT in PubMed, Embase, and the Cochrane Library from study inception to May 23, 2022, without language restrictions. The keywords included disability, IVT, and AIS. The complete search algorithm is described in Supporting Information 2. We also conducted a manual search of all included studies and related review articles to avoid the possibility of missing any eligible studies.

### 2.2 Study selection and data extraction

We investigated the safety and efficacy of IVT in AIS patients with PSD. We defined disability according to the Rankin Focused Assessment (Saver et al., 2010) and divided the disability into PSD (defined as modified Rankin Scale [mRS] score ≥2), severe disability (defined as mRS score = 5), and death (defined as mRS score = 6). Criteria for study selection: (1) articles that included patients with AIS treated with IVT, (2) articles that identified the association of the presence or absence of PSD with safety and efficacy outcomes, (3) articles on adult patients (>18 years old), and (4) article language was English. Studies with less than 50% of patients receiving IVT, or study types of case reports, reviews, letters, and meta‐analyses, were excluded.

Two independent investigators (Xinting Wu and Qiangji Bao) reviewed all the retrieved articles and extracted data. A third investigator (Mingfei Yang) was consulted if there was any disagreement. The study characteristics were extracted, including the name of the first author, year of publication, study design, mean age, country, sex distribution, IVT drugs, and primary endpoint (24 h NIHSS improvement, sICH, death, etc.). Data extraction is summarized in Table 1. The odds ratios (ORs) or original data were extracted in each study.

**TABLE 1:** Characteristics of included studies.

			age(year)		Sample size		Gender female %					
First author and year of publication	Country	Study type	nPD	PD	nPD	PD	nPD	PD	Eligibility	Thrombolysis	Follow‐up	Primary endpoint
Cooray et al. (2021)	Sweden	Prospective	71 [61–78]	78 [69–85]	83,451	4559	43.7%	57.7%	Ischemic stroke patients treated with intravenous thrombolysis	Alteplase	2003–2017	24 h NIHSS improvement >4, sICH, 7‐day death
Caruso et al. (2020)	Italy	Retrospective	NR	87 (45–95)	NR	35	NR	74.3%	Ischemic stroke pre‐existing disability (mRS≥2), rt‐PA treatment	rt‐PA	2015–2017	sICH, mortality, mRS score
Cooray et al. (2020)	Sweden	Prospective	71 [61–78]	78 [69–85]	83,451	4559	43.7%	57.7%	Ischemic stroke patients treated with intravenous thrombolysis	Alteplase	2003–2017	24 h NIHSS improvement >4, sICH, PH, 7‐day death
Gumbinger et al. (2019)	Germany	Retrospective	77.2 (10.6)	80.9 (9.1)	40,335	12,406	46.2%	58.2%	Ischemic stroke patients	IVT	2008–2014	Favorable functional outcome, mortality
Goldhoorn et al. (2018)	Poland	Prospective	69 (59–78)	80 (71–86)	1284	157	45%	59%	Ischemic stroke patients	IVT	2014–2016	Favorable functional outcome, Sich, mortality at 3 months, 24 h NIHSS improvement >4
Zhang et al. (2018)	Australia	Retrospective	71.8 (13.1)	82.1 (8.7)	680	140	42.1%	59.3%	Ischemic stroke patients	Alteplase	2005–2016	Favorable functional outcome, mortality at 3 months, sICH
Gensicke et al. (2016)	Europe	Prospective	71 (60–79)	84 (77–88)	6941	489	57.4%	33.9%	Ischemic stroke patients	IVT	2003–2014	Favorable functional outcome, mortality, sICH
Karlinski et al. (2014)	Europe (10 countries)	Prospective	72 (64–78)	75 (68–81)	6780	464	42%	53%	Ischemic stroke patients	rt‐PA, alteplase	2003–2011	Favorable functional outcome, mortality at 3 months, Sich, 24 h NIHSS improvement >4
Karlinski et al. (2013)	Eastern Europe	Prospective	69 (61–78)	75 (67–83)	6786	464	NR	NR	Ischemic stroke patients	Alteplase	2003–2011	sICH, mortality at 3 months
Foell et al. (2003)	Canada	Retrospective	71 (9)	76 (8)	88	24	NR	NR	Ischemic stroke patients	rt‐PA	3 month	Favorable functional outcome, mortality at 3 months

Abbreviations: IVT, intravenous thrombolysis; NR, no report; PD, prestroke disability; PH, parenchymal hemorrhage; rt‐PA, recombinant tissue plasminogen activator; sICH, symptomatic intracerebral hemorrhage.

### 2.3 Outcomes

We evaluated the unadjusted and adjusted efficacy outcomes, including favorable functional outcome (FFO), which was defined as an mRS score of 0–1 or a return to the pre‐stroke mRS score, sICH, 24 h NIHSS improvement according to the various definitions used in the included studies (Supporting Information 3), and mortality.

Studies reporting data on PSD outcomes with adjusted and unadjusted data were assessed separately. Additional analyses were conducted after pooling available data for 24 h NIHSS improvement, sICH, mortality, and FFO definitions for patients with different mRS scores.

### 2.4 Risk of bias assessment

Two independent investigators (Xinting Wu and Yiming Li) performed quality control and bias identification, and a third investigator (Mingfei Yang) resolved potential disagreements.

### 2.5 Data synthesis and statistical analysis

The ORs, standardized mean differences, and their corresponding 95% confidence intervals (CIs) were calculated to measure the effect size and evaluate the association of PSD (vs. no PSD) with safety and efficacy outcomes among AIS patients treated with IVT.

For the qualitative interpretation of heterogeneity, *I* ^2^ > 50% and *I* ^2^ > 75% were considered substantial and considerable heterogeneity, respectively (Cumpston et al., 2019). The funnel plot was used to evaluate publication bias across individual studies. The funnel plot asymmetry was assessed using the Egger linear regression test, and the significance level was *p* < .10. We used a random‐effects model to calculate the pooled ORs in both the overall and subgroup analyses. An equivalent *z*‐test was performed for each pooled OR, and a 2‐tailed value of *p* < .05 was considered statistically significant.

All statistical analyses were conducted with the Comprehensive Meta‐Analysis (CMA2.0) and Cochrane Collaboration's Review Manager Software Package (RevMan5.3).

## 3 RESULTS

### 3.1 Study selection and study characteristics

**FIGURE 1:**
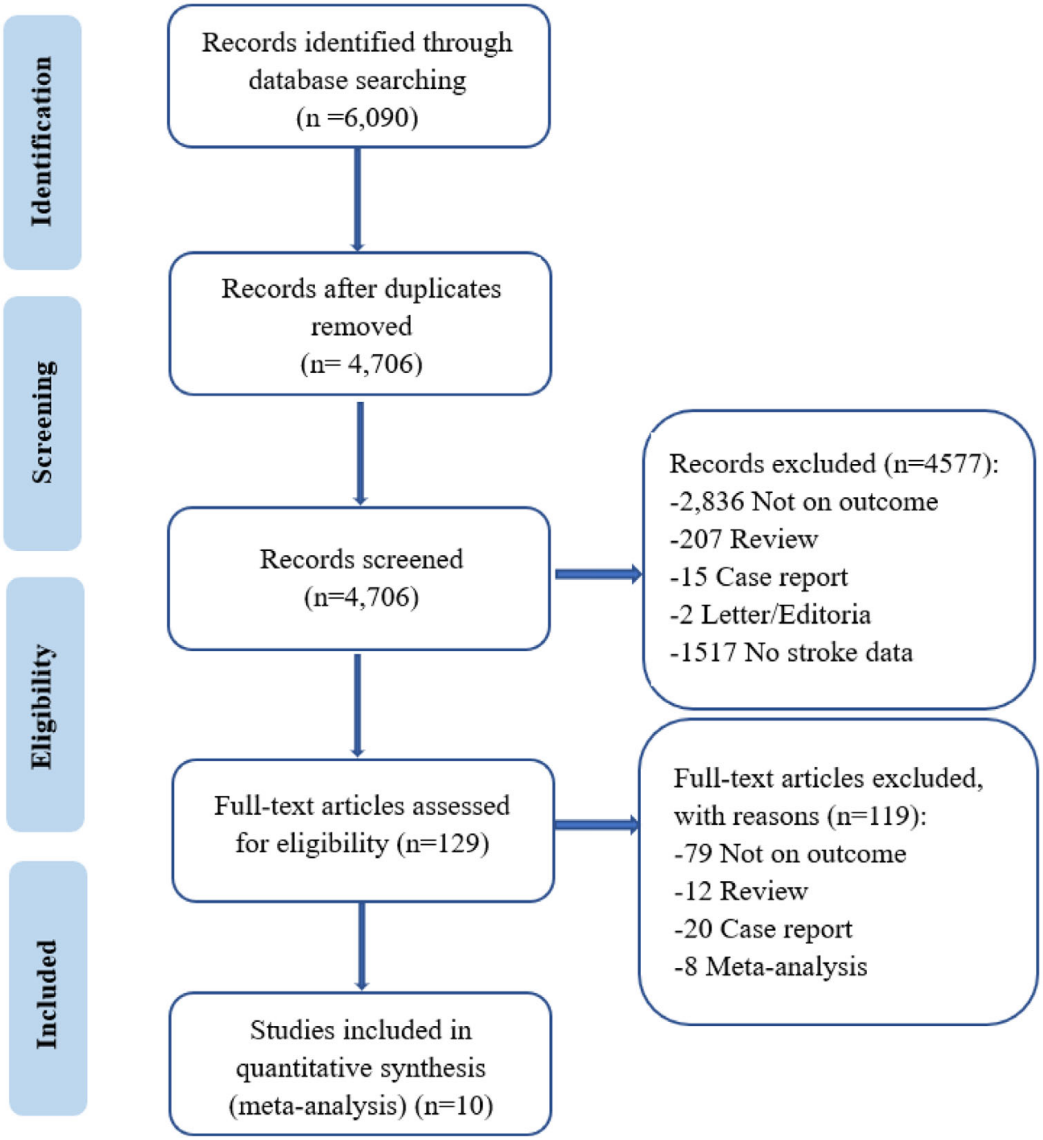
Flow chart of literature search and study selection.

The flowchart of the study selection is shown in Figure 1. Initially, 6090 potentially relevant articles were identified. After the removal of duplicate publications and after screening titles or abstracts, 10 potentially relevant articles were subjected to full‐text review. Finally, 10 studies involving 245,773 participants were included in the meta‐analysis, including 6 prospective (Cooray et al., 2020, 2021; Gensicke et al., 2016; Goldhoorn et al., 2018; Karlinski et al., 2013, 2014) and 4 retrospective (Caruso et al., 2020; Foell et al., 2003; Gumbinger et al., 2019; Zhang et al., 2018) observational studies. Table 1 shows the study design and the characteristics of included studies. The baseline characteristics of the included patients in the comparison arms (PSD vs. no PSD) were presented. The studies were conducted in Sweden (*n* = 2), Italy (*n* = 1), Germany (*n* = 1), Poland (*n* = 1), Australia (*n* = 1), Europe (*n* = 3), and Canada (*n* = 1). The association of PSD versus no PSD with safety and efficacy outcomes was reported in these studies. Statistically, the age of the patients with PSD was significantly higher than that of patients without PSD.

### 3.2 Study quality and publication bias

The Newcastle–Ottawa scale assessed the risk of bias in cohort studies. The risk of outcome bias was considered moderate. The detailed confounders that were included in the adjusted analyses of available studies are displayed in Supporting Information 4. The overall score of the Newcastle–Ottawa scale was considered to represent an overall high quality. The detailed study quality is displayed in Supporting Information 5. Analysis with funnel plot revealed no evidence of asymmetry in studies reporting the unadjusted outcomes. The Egger test was not applicable because of the small number of studies (<10).

### 3.3 Association between PSD and outcomes

Table 2 provides an overview of the overall unadjusted and adjusted analyses investigating the association of PSD with various clinical outcomes.

**TABLE 2:** Overview of the safety and efficacy analyses on different endpoints.

	Unadjusted analyses				Adjusted analyses			
Outcome	Studies, *n*	OR (95% CI)	*p* Value	Heterogeneity (*I* ^2^) (%)	Studies, *n*	OR (95% CI)	*p* Value	Heterogeneity (*I* ^2^) (%)
sICH	9	.773 (.481–1.243)	.288	85.687	5	.857 (.725–1.012)	.069	35.309
sICH (mRS = 2–5)	2	.480 (.099–2.324)	.362	52.532	1	.780 (.615–.989)	.040	0
sICH (mRS = 3–5)	7	.810 (.492–1.334)	.408	87.103	4	.939 (.743–1.188)	.601	28.994
24 h NIHSS improvement	5	.867 (.819–.917)	.000	27.991	5	.837 (.799–.876)	.000	48.073
24 h NIHSS improvement (mRS = 2–5)	2	.870 (.822–.922)	.000	0	2	.871 (.822–.922)	.000	0
24 h NIHSS improvement (mRS = 3–5)	3	.781 (.593–1.028)	.078	61.479	3	.779 (.722–.841)	.000	.539
FFO	7	1.057 (1.015–1.100)	.007	94.951	5	1.087 (1.002–1.179)	.044	0
FFO (mRS = 2–5)	2	1.059 (1.017–1.103)	.006	0	2	1.121 (1.028–1.222)	.010	0
FFO (mRS = 3–5)	5	.847 (0.551–1.301)	.448	95.104	3	.866 (.684–1.096)	.232	0
Mortality	10	1.793 (1.336–2.407)	.000	91.640	7	1.789 (1.413–2.264)	.000	80.904
Mortality (mRS = 2–5)	2	1.648 (.678–4.002)	.270	62.888	2	1.343 (.803–2.248)	.261	26.309
Mortality (mRS = 3–5)	8	1.812 (1.327–2.476)	.000	93.326	5	1.930 (1.481–2.515)	.000	72.665
In‐hospital mortality	3	1.147 (.875–1.504)	.321	95.775	2	1.291 (1.120–1.488)	.000	60.8
90day mortality	5	2.349 (1.397–3.948)	.001	85.268	4	2.170 (1.692–2.783)	.000	0

Abbreviations: FFO, favorable functional outcome; mRS, modified Rankin Scale; OR, odds ratio; sICH, symptomatic intracranial hemorrhage.

#### 3.3.1 Unadjusted analysis

Among patients with AIS treated with IVT, PSD was associated with higher odds of pooled mortality (10 studies; OR 1.793, 95% CI, 1.336–2.407, *p* = .000, *I* ^2^ = 91.640%; Figure 2.1) and FFO (7 studies; OR 1.057, 95% CI, 1.015–1.100, *p* = .007, *I* ^2^ = 94.951%; Figure 2.2). There was no significant association for a history of PSD and odds of 24 h NIHSS improvement (five studies; OR .867, 95% CI, .819–.917, *p* = .000, *I* ^2^ = 27.991%; Figure 2.3) and sICH (nine studies; OR .773, 95% CI, .481–1.243, *p* = .288, *I* ^2^ = 85.687%; Figure 2.4). Significant heterogeneity (*I* ^2^ > 50%) was found in all the outcomes in the unadjusted analyses except for 24 h NIHSS improvement. Funnel plots (Supporting Information 6) and tests for publication bias showed non‐significant results.

FIGURE 2Forest plots with pooled unadjusted odds ratio from a random‐effects model in patients with versus without premorbid disability (2.1: mortality; 2.2: favorable function outcome; 2.3: 24 h NIHSS improvement; 2.4: symptomatic intracerebral hemorrhage) (The area of each square is proportional to the inverse of the variance of the estimated log odds ratio [OR]. Diamonds represent point estimates of OR, and horizontal lines represent 95% confidence intervals [CIs]. The open diamonds represent the combined OR for each subgroup. The solid line represents OR = 1).

**Figure:**
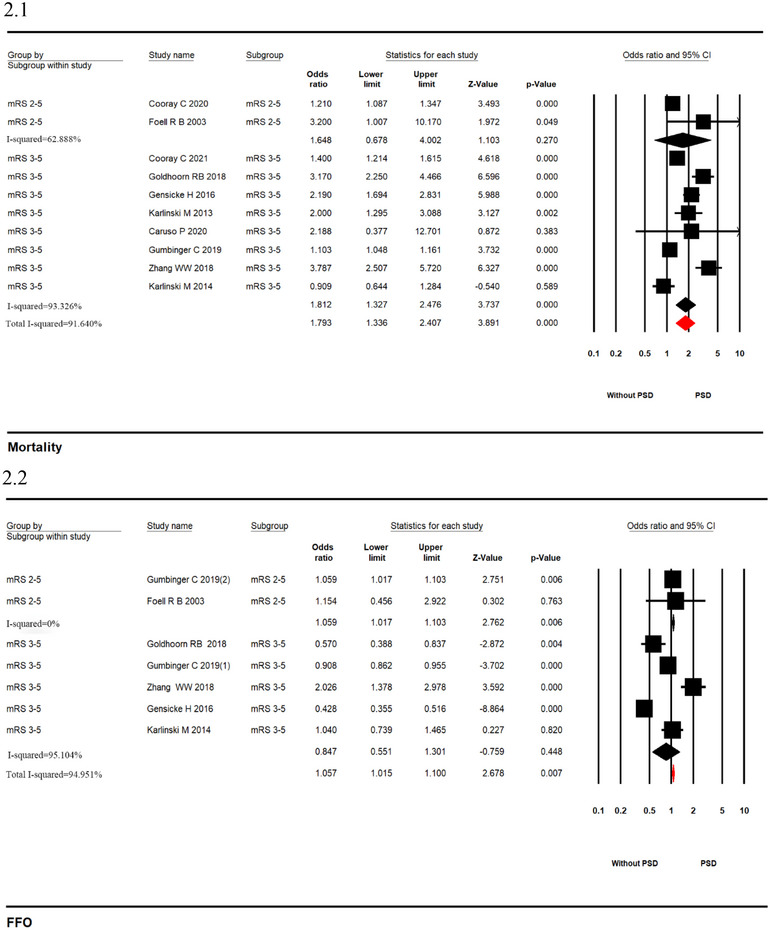

**Figure:**
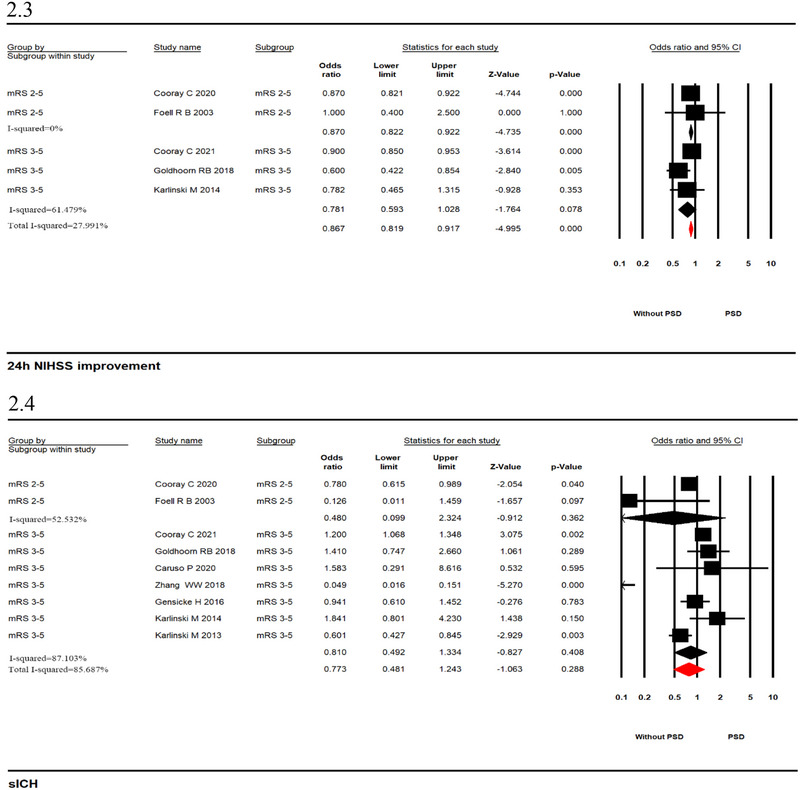

#### 3.3.2 Adjusted analysis

In adjusted analyses, PSD was associated with increased odds of mortality (seven studies; OR_adj_ 1.789, 95% CI, 1.413–2.264, *p* = .000, *I* ^2^ = 80.904%; Figure 3.1) and FFO (five studies; OR_adj_ 1.087; 95% CI, 1.002–1.179, *p* = .044, *I* ^2^ = 0%; Figure 3.2). No significant associations were observed for 24 h NIHSS improvement (five studies; OR_adj_ .837, 95% CI, .799–0.876, *p* = .000, *I* ^2^ = 48.073%; Figure 3.3) and sICH (five studies; OR_adj_ .857, 95% CI, .725–1.012, *p* = .069, *I* ^2^ = 35.309%; Figure 3.4). Analysis of adjusted data found that heterogeneity was significantly reduced.

FIGURE 3Forest plots with pooled adjusted odds ratio from a random‐effects model in patients with versus without prostroke disability (3.1: mortality; 3.2: favorable function outcome; 3.3: 24 h NIHSS improvement; 3.4: symptomatic intracerebral hemorrhage [sICH]).

**Figure:**
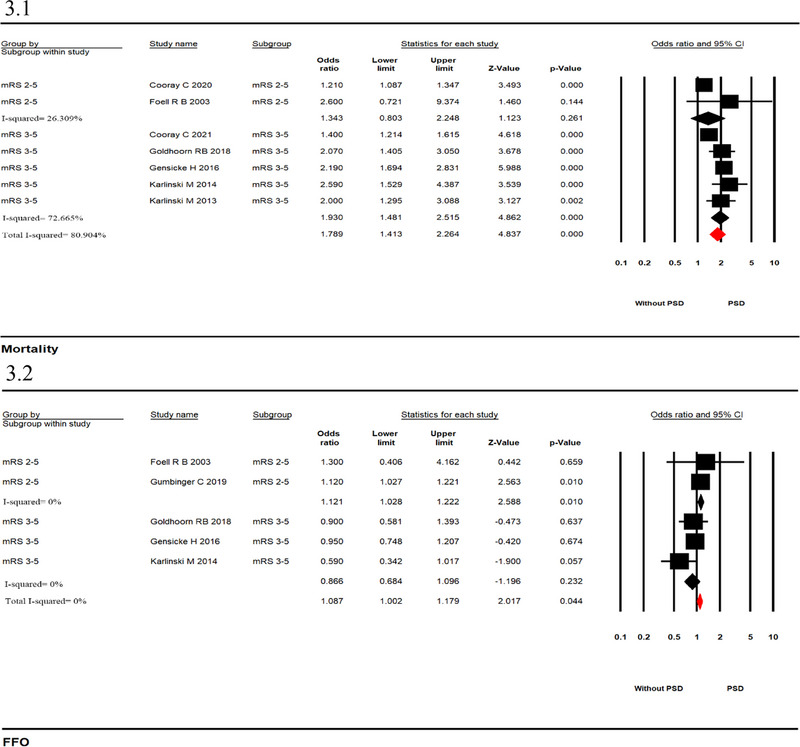

**Figure:**
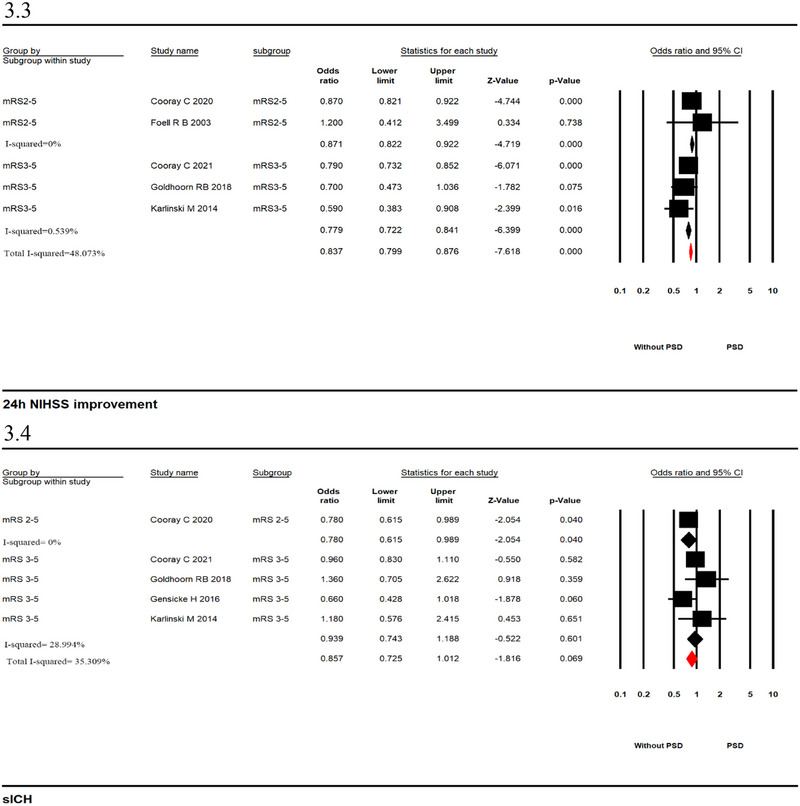

Interestingly, we might have stumbled across a study (Merlino et al., 2019) that provides an overview of the outcome between PSD with IVT and without. PSD with IVT was related to an increased likelihood of favorable outcome (defined as a return to the pre‐stroke mRS score at 3 months after AIS) (OR 7.26, 95% CI, 2.51–21.02, *p* = .001) and major neurological improvement (defined as an improvement of ≥8 points on the NIHSS from baseline or an NIHSS score of 0 at discharge) (OR 3.7, 95% CI, 1.32–10.35, *p* = .001). Importantly, the prevalence of 3‐month mortality, ICH, and sICH did not significantly differ between the two groups.

### 3.4 Prevalence of mortality and outcome with different mRS scores

**FIGURE 4:**
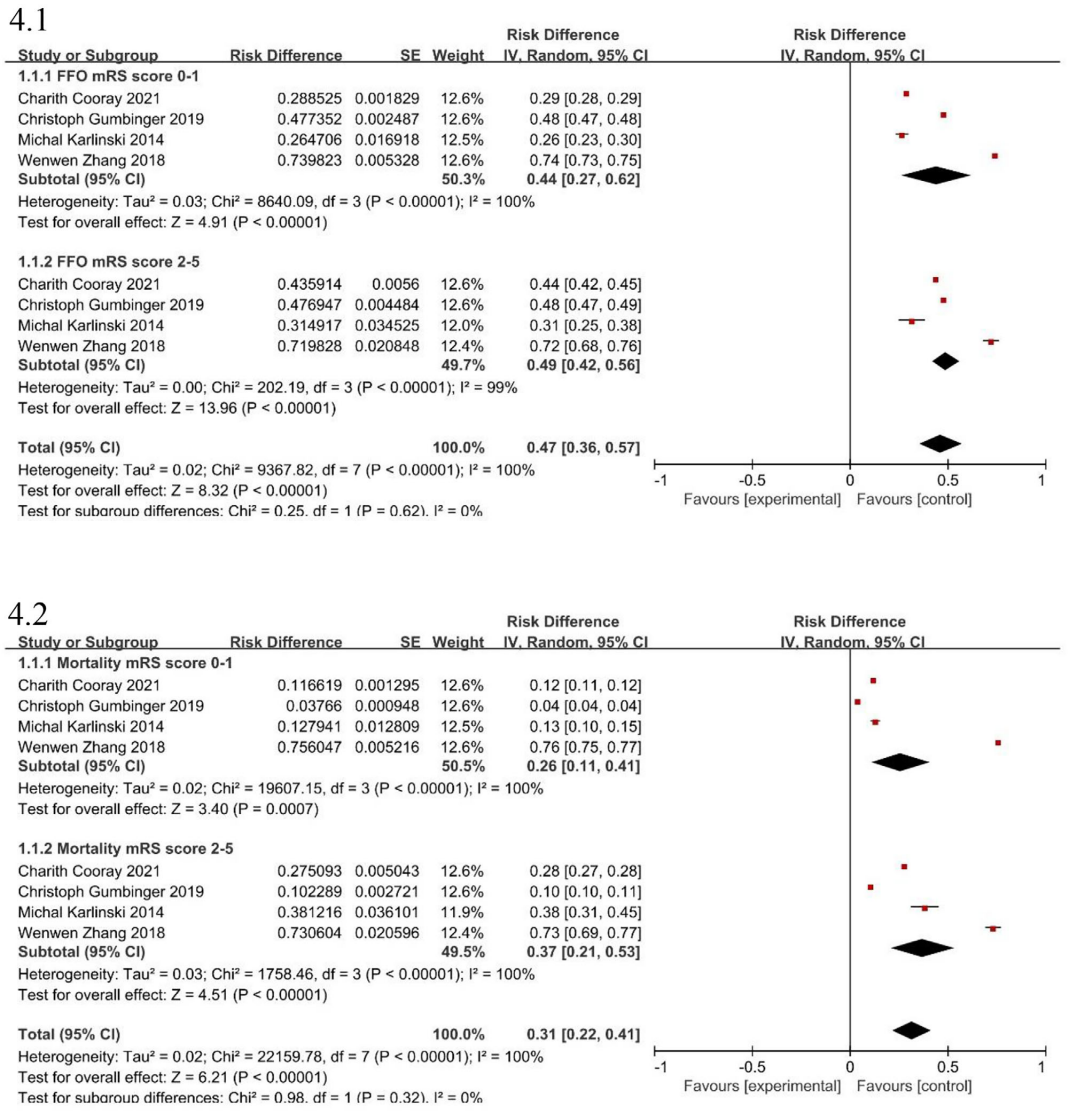
Forest plots showing pooled prevalence of outcome after intravenous thrombolysis (IVT) in acute ischemic stroke (AIS) patients with disability (4.1: favorable outcome; 4.2: death).

The prevalence of mortality, a favorable outcome, and 95% CI in different mRS scores (mRS score of 0–1: without PSD; mRS score of 2–5: PSD) were calculated separately for each study. The sample size and published years could also be found (Figure 4). The FFO and mortality prevalence ranged, as shown in Figure 4.1 (mRS score = 0–1: 44%; 95% CI, .27–.62; mRS score = 2–5: 49%; 95% CI, .42–.56) and Figure 4.2 (mRS score = 0–1: 26%; 95% CI, .11–.41; mRS score = 2–5: 37%; 95% CI, .21–.53). The heterogeneity of the analysis was high.

### 3.5 Subgroup analysis

We also evaluated those studies based on different definitions of PSD (defined as pre‐stroke mRS score = 2–5 or mRS score = 3–5). We found that mortality (mRS score 2–5, two studies; OR_adj_ 1.343, 95% CI, .803–2.248, *p* = .261, *I* ^2^ = 26.309%; mRS score 3–5, five studies; OR_adj_ 1.930, 95% CI, 1.481–2.515, *p* = .000, *I* ^2^ = 72.665%; Figure 3.1) and FFO (mRS score 2–5, two studies; OR_adj_ 1.121, 95% CI, 1.028–1.222, *p* = .010, *I* ^2^ = 0%; mRS score 3–5, three studies; OR_adj_ .866, 95% CI, .684–1.096, *p* = .232, *I* ^2^ = 0%; Figure 3.2) were different between the two groups under different PSD definitions. Unfortunately, no statistical significance was observed for lower odds of pooled mortality in patients with an mRS score of 2–5. Meanwhile, the ORs of pooled 24 h NIHSS improvement and sICH did not significantly differ between the two groups. The heterogeneity of the analysis decreased (24 h NIHSS improvement: mRS score 2–5, two studies; OR_adj_ .871, 95% CI, .822–.922, *p* = .000, *I* ^2^ = 0%; mRS score 3–5, three studies; OR_adj_ .779, 95% CI, .722–.841, *p* = .000, *I* ^2^ = .539%; Figure 3.3. sICH: mRS score 2–5, one study; OR_adj_ .780, 95% CI, .615–.989, *p* = .040, *I* ^2^ = 0%; mRS score 3–5, four studies; OR_adj_ .939, 95% CI, .743–1.188, *p* = .601, *I* ^2^ = 28.994%; Figure 3.4). In addition, we further analyzed subgroups of time to death using adjusted data and found an association among early in‐hospital mortality, 90‐day mortality, and PSD. The heterogeneity of 90‐day mortality was 0%, and in‐hospital mortality was 60.8% (Supporting Information 7).

### 3.6 Sensitivity analyses

We performed additional analyses for the adjusted associations, stratified by study types (prospective vs. retrospective studies). A retrospective study was removed. No significant difference was found in the adjusted associations of PSD with the likelihood of sICH, FFO, 24 h NIHSS improvement, and mortality. We removed a study when analyzing mortality. Heterogeneity (*I* ^2^ = 28%) was observed between the studies, and the results indicated a statistically significant difference. The heterogeneity of 24 h NIHSS improvement and sICH was reduced, which was 0% and 12%, respectively. The results indicated a statistically significant difference. We believe that the results of the removed articles may be different due to the different study designs, which can lead to differences in results (Supporting Information 8). Additionally, we plotted a histogram to compare with the forest map (Supporting Information 9).

## 4 DISCUSSION

The initial objective of this study was to identify the safety and efficacy of IVT among AIS patients with PSD. A total of 245,773 participants with AIS treated with IVT were included in this systematic review and meta‐analysis. We found that compared with patients without PSD, patients with PSD had higher odds of FFO (defined as an mRS score of 0–1 or a return to the pre‐stroke mRS score) (OR_adj_ 1.087, *p* = .044), similar likelihood of sICH (OR_adj_ .857, *p* = .069) and 24 h NIHSS improvement (OR_adj_ .837, *p* = .000), but higher mortality rate (OR_adj_ 1.789, *p* = .000). These findings further supported the application of IVT in AIS patients with PSD.

A recent study has shown that IVT is safe for patients with PSD (Beland et al., 2022). However, we found that the result of the adjusted data analysis was not statistically significant, and the heterogeneity was high. Thus, the results were not convincing. There was also no clear distinction in the definition of PSD (e.g., pre‐stroke mRS score = 2–5 or mRS score = 3–5). Therefore, our study included 10 studies, including prospective and retrospective studies. We performed a comprehensive evaluation, including sensitivity and subgroup analyses by the definition of disability in the included articles and an assessment of various safety and efficacy outcomes associated with PSD. The results showed statistical significance.

In this meta‐analysis, patients with PSD who received IVT were more likely to achieve FFO than patients without PSD. This might be related to the fact that patients who have just developed a stroke have more difficulty returning to an mRS score of 0 or 1 after a stroke. Namely, a large proportion of patients with PSD and mRS scores of 3–5 achieved FFO, although they still had an mRS score of ≥3. Patients without PSD needed an mRS score of 0–2 to achieve FFO. We also found that an mRS score of 2–5 was associated with a lower mortality rate after IVT, which is interesting and may be related to different definitions of disability before stroke onset. We considered whether a large proportion of patients with an mRS score of 2 have lower mortality, thus affecting the final results. Although the results were not statistically significant, considering the small sample size of the included studies, there may be some confounding factors (such as study design, inclusion, and exclusion criteria). This should be further investigated. For patients with an mRS score of 2, clinicians may prefer IVT. Thus, it may be reasonable for patients with PSD to maintain their premorbid status to be considered to have a favorable outcome. In addition, the guidelines suggest that thrombolysis/thrombectomy may be justified in certain cases where treatment may be associated with less neurological improvement and higher mortality (Powers et al., 2018). In this regard, it is encouraging that IVT can facilitate better recovery and improve long‐term clinical and health economic outcomes in such patients. However, this has not been validated by health economic data from registries or trials.

A study involving sequential hospital admissions with acute stroke has demonstrated that every point increase in the pre‐stroke mRS score was associated with an increase in the rate of length‐of‐stay, discharge destination, mortality, and complications (Quinn et al., 2017). In general, IVT is not considered the first choice for AIS patients with PSD before stroke because of their possibly high mortality rate (Quinn et al., 2017; Salwi et al., 2021). Previous studies have demonstrated increased odds of mortality, whereas there was no significant difference between patients with and without PSD in return to their pre‐stroke level of function, sICH, or any ICH (Gensicke et al., 2016; Karlinski et al., 2014). In this study, we also found that PSD was associated with the risk of in‐hospital mortality and 90‐day mortality. However, it is a fact that patients with moderate or severe premorbid disabilities represent a very small proportion of the total AIS patients receiving IVT. Most studies are limited by insufficient sample size, which may lead to adverse outcomes. Furthermore, by comparing the data from the enrolled population, we found that PSD patients who received IVT were, on average, older than those who did not. We stumbled upon a cohort study that investigated the outcome of patients with PSD who received IVT or not (Merlino et al., 2019), which suggests that IVT has a good functional outcome. Our study further confirmed the reliability and safety of IVT in AIS patients with PSD.

Interestingly, however, US guidelines indicate that the risk of sICH in patients with PSD after thrombolysis does not seem to increase. The updated guidelines on the early management of ischemic stroke by the American Heart Association (Powers et al., 2019) include eligibility recommendations for IVT for patients with AIS and PSD. However, the guidelines also caution that premorbid disabilities do not seem to independently increase the risk of sICH. Our findings were consistent with the guidelines. Because the safety and efficacy of IVT among these patients remain largely unknown, the guidelines recommend that IVT decision‐making should consider the factors of quality of life, social support, place of residence, and so on. The above‐mentioned recommendations were verified in our comprehensive systematic review and meta‐analysis. Our results may also guide clinicians to individualize IVT decisions and weigh the risks and benefits for AIS patients with PSD. Our findings provide further evidence and underscore increased safety concerns about IVT in patients with PSD. Multicenter randomized controlled trials are needed to validate our findings.

We unexpectedly found that lacunar stroke accounted for one quarter of all cerebral infarction, and its pathophysiology, prognosis, and clinical characteristics were different from those of other types of cerebral infarction. Lacunar stroke is an adverse vascular disease with a high risk of medium‐ and long‐term recurrences and vascular dementia (Arboix et al., 2014). Therefore, the relationship between the safety and efficacy of IVT in PSD patients with lacunar or non‐lacunar AIS should be further investigated.

There are some limitations of this study. First, the majority of our included studies were observational studies with retrospective design, which might have predisposes to inherent biases. Furthermore, we were unable to control for mitigating effects of potential confounding variables because these relationships could be assessed only in the setting of individual patient data pooling large datasets from numerous stroke registries. Second, some studies did not report mRS scores as per our predefined disability cut‐points. Accordingly, we subdivided the patients according to the nearest mRS cut‐points and categorized them as having mild, moderate, or severe disabilities, which could potentially introduce sampling bias. Third, the limited available data included in our study might moderate the association of IVT with safety or efficacy outcomes in patients with AIS with PSD.

Future multicenter randomized controlled trials should focus on the effect modifiers affecting the safety and efficacy of IVT treatment in PSD, such as the dose, timing, and administration speed of IVT (Lee et al., 2016). Based on these assumptions, it may be worthwhile to further explore how treatment modifications, such as the administration of lower doses of IVT and tighter blood pressure management, can enhance the safety of IVT in patients with PSD without significantly limiting its efficacy. Guidelines (Turc et al., 2022) suggest that patients with anterior circulation large‐vessel occlusion stroke should receive IVT in addition to mechanical thrombectomy if the two treatments are not contraindicated. Whether PSD is contraindicated should be further studied.

## 5 CONCLUSIONS

Our systematic review and meta‐analysis showed that although a history of PSD was independently associated with higher mortality in AIS patients treated with IVT, no significant difference was found in other adverse outcomes between AIS patients with and without PSD who underwent IVT. Physicians should weigh the risks and benefits of IVT for AIS patients with PSD. Our findings may provide further evidence and highlight safety concerns about IVT in patients with PSD who are not routinely excluded from IVT.

## AUTHOR CONTRIBUTIONS

**Qiangji Bao**: Data curation; methodology; software; writing—original draft; writing—review and editing. **Xinting Wu**: Data curation; visualization; writing—original draft. **Yiming Li**: Validation. **Shujun Chen**: Validation. **Qiang Zhang**: Validation. **Peng Yang**: Validation. **Mingfei Yang**: Supervision.

## REVIEWER DISCLOSURES

Peer reviewers on this manuscript have no relevant financial or other relationships to disclose.

## CONFLICT OF INTEREST STATEMENT

The authors declare that there are no conflicts of interest regarding the publication of this article.

### PEER REVIEW

The peer review history for this article is available at https://publons.com/publon/10.1002/brb3.3431.

## Supporting information

Supporting Information

## Data Availability

All data generated or analyzed during this study were included in this article (and/or) its supporting information files. Further inquiries could be directed to the corresponding author.

## References

[brb33431-bib-0001] Arboix, A. , Blanco‐Rojas, L. , & Martí‐Vilalta, J. L. (2014). Advancements in understanding the mechanisms of symptomatic lacunar ischemic stroke: Translation of knowledge to prevention strategies. Expert Review of Neurotherapeutics, 14(3), 261–276. 10.1586/14737175.2014.884926 24490992

[brb33431-bib-0002] Beland, B. , Bala, F. , & Ganesh, A. (2022). Thrombolysis for acute ischemic stroke in patients with premorbid disability: A meta‐analysis. Stroke; A Journal of Cerebral Circulation, 53(10), 3055–3063. 10.1161/STROKEAHA.121.038374 35686556

[brb33431-bib-0003] Berge, E. , Whiteley, W. , Audebert, H. , De Marchis, G. M. , Fonseca, A. C. , Padiglioni, C. , Pérez De La Ossa, N. , Strbian, D. , Tsivgoulis, G. , & Turc, G. (2021). European Stroke Organisation (ESO) guidelines on intravenous thrombolysis for acute ischaemic stroke. European Stroke Journal, 6(1), I–LXII. 10.1177/2396987321989865 PMC7995316 33817340

[brb33431-bib-0004] Caruso, P. , Ajčević, M. , Furlanis, G. , Ridolfi, M. , Lugnan, C. , Cillotto, T. , Naccarato, M. , & Manganotti, P. (2020). Thrombolysis safety and effectiveness in acute ischemic stroke patients with pre‐morbid disability. Journal of Clinical Neuroscience, 72, 180–184. 10.1016/j.jocn.2019.11.047 31875830

[brb33431-bib-0005] Cooray, C. , Karlinski, M. , Kobayashi, A. , Ringleb, P. , Kõrv, J. , Macleod, M. J. , Dixit, A. , Azevedo, E. , Bladin, C. , & Ahmed, N. (2020). Safety and efficacy of intravenous thrombolysis in acute ischaemic stroke patients with prestroke disabilities. International Journal of Stroke, 15(Suppl 1), 198. 10.1177/1747493020954605 32878588

[brb33431-bib-0006] Cooray, C. , Karlinski, M. , Kobayashi, A. , Ringleb, P. , Kõrv, J. , Macleod, M. J. , Dixit, A. , Azevedo, E. , Bladin, C. , & Ahmed, N. (2021). Safety and early outcomes after intravenous thrombolysis in acute ischemic stroke patients with prestroke disability. International Journal of Stroke, 16(6), 710–718. 10.1177/1747493020954605 32878588

[brb33431-bib-0007] Cumpston, M. , Li, T. J. , Page, M. J. , Chandler, J. , Welch, V. A. , Higgins, J. P. , & Thomas, J. (2019). Updated guidance for trusted systematic reviews: A new edition of the Cochrane Handbook for Systematic Reviews of Interventions. Cochrane Database of Systematic Reviews (Online), 10, ED000142. 10.1002/14651858.ED000142 PMC10284251 31643080

[brb33431-bib-0008] Emberson, J. , Lees, K. R. , Lyden, P. , Blackwell, L. , Albers, G. , Bluhmki, E. , Brott, T. , Cohen, G. , Davis, S. , Donnan, G. , Grotta, J. , Howard, G. , Kaste, M. , Koga, M. , Von Kummer, R. , Lansberg, M. , Lindley, R. I. , Murray, G. , Olivot, J. M. , … Hacke, W. (2014). Effect of treatment delay, age, and stroke severity on the effects of intravenous thrombolysis with alteplase for acute ischaemic stroke: A meta‐analysis of individual patient data from randomised trials. Lancet, 384(9958), 1929–1935. 10.1016/S0140-6736(14)60584-5 25106063 PMC4441266

[brb33431-bib-0009] Foell, R. B. T. , Silver, B. , Merino, J. G. , Wong, E. H. , Demaerschalk, B. M. , Tamayo, F. P. A. , & Hachinski, V. (2003). Effects of thrombolysis for acute stroke in patients with pre‐existing disability. Canadian Medical Association Journal, 169(3), 193–197. 12900476 PMC167119

[brb33431-bib-0010] Gensicke, H. , Strbian, D. , Zinkstok, S. M. , Scheitz, J. F. , Bill, O. , Hametner, C. , Moulin, S. , Zini, A. , Kägi, G. , Pezzini, A. , Padjen, V. , Béjot, Y. , Corbiere, S. , Zonneveld, T. P. , Seiffge, D. J. , Roos, Y. B. , Traenka, C. , Putaala, J. , Peters, N. , … Engelter, S. T. (2016). Intravenous thrombolysis in patients dependent on the daily help of others before stroke. Stroke; A Journal of Cerebral Circulation, 47(2), 450–456. 10.1161/STROKEAHA.115.011674 26797662

[brb33431-bib-0011] Goldhoorn, R. J. B. , Verhagen, M. , Dippel, D. W. J. , Van Der Lugt, A. , Lingsma, H. F. , Roos, Y. B. W. E. M. , Majoie, C. B. L. M. , Vos, J. A. , Boiten, J. , Van Zwam, W. H. , Van Oostenbrugge, R. J. , & Van Den Wijngaard, I. (2018). Safety and outcome of endovascular treatment in prestroke‐dependent patients. Stroke; A Journal of Cerebral Circulation, 49(10), 2406–2414. 10.1161/STROKEAHA.118.022352 30355090

[brb33431-bib-0012] Gumbinger, C. , Ringleb, P. , Ippen, F. , Ungerer, M. , Reuter, B. , Bruder, I. , Daffertshofer, M. , & Stock, C. (2019). Outcomes of patients with stroke treated with thrombolysis according to prestroke Rankin Scale scores. Neurology, 93(20), e1834–e1843. 10.1212/WNL.0000000000008468 31653709

[brb33431-bib-0013] Hacke, W. , Kaste, M. , Bluhmki, E. , Brozman, M. , Dávalos, A. , Guidetti, D. , Larrue, V. , Lees, K. R. , Medeghri, Z. , Machnig, T. , Schneider, D. , Von Kummer, R. , Wahlgren, N. , & Toni, D. (2008). Thrombolysis with alteplase 3 to 4.5 hours after acute ischemic stroke. New England Journal of Medicine, 359, 1317–1329. 10.1056/NEJMoa0804656 18815396

[brb33431-bib-0014] Karlinski, M. , Kobayashi, A. , Czlonkowska, A. , Mikulik, R. , Vaclavik, D. , Brozman, M. , Švigelj, V. , Csiba, L. , Fekete, K. , Kõrv, J. , Demarin, V. , Vilionskis, A. , Jatuzis, D. , Krespi, Y. , Ahmed, N. , Wahlgren, N. , Mikulik, R. , Czlonkowska, A. , Brozman, M. , … Shamalov, N. (2014). Role of preexisting disability in patients treated with intravenous thrombolysis for ischemic stroke. Stroke; A Journal of Cerebral Circulation, 45(3), 770–775. 10.1161/STROKEAHA.113.003744 24496395

[brb33431-bib-0015] Karlinski, M. A. , Kobayashi, A. , Onkowska, A. , Mikulik, R. , Brozman, M. , Svigelj, V. , Csiba, L. , Fekete, K. , Korv, J. , Demarin, V. , & Jatuzis, D. (2013). The role of pre‐existing disability in patients treated with intravenous thrombolysis for acute stroke. Cerebrovascular Diseases, 35(Suppl 3), 170.

[brb33431-bib-0016] Lansberg, M. G. , Albers, G. W. , & Wijman, C. A. C. (2007). Symptomatic intracerebral hemorrhage following thrombolytic therapy for acute ischemic stroke: A review of the risk factors. Cerebrovascular Diseases, 24(1), 1–10. 10.1159/000103110 17519538

[brb33431-bib-0017] Lee, S. H. , Kim, B. J. , Han, M. K. , Park, T. H. , Lee, K. B. , Lee, B. C. , Yu, K. H. , Oh, M. S. , Cha, J. K. , Kim, D. H. , Nah, H. W. , Lee, J. , Lee, S. J. , Ko, Y. , Kim, J. G. , Park, J. M. , Kang, K. , Cho, Y. J. , Hong, K. S. , … Bae, H. J. (2016). Should we exclude acute stroke patients with previous intracerebral hemorrhage from receiving intravenous thrombolysis? International Journal of Stroke, 11(7), 783–790. 10.1177/1747493016654289 27312681

[brb33431-bib-0018] Merlino, G. , Corazza, E. , Lorenzut, S. , Gigli, G. , Cargnelutti, D. , & Valente, M. (2019). Efficacy and safety of intravenous thrombolysis in patients with acute ischemic stroke and pre‐existing disability. Journal of Clinical Medicine, 8(3), 400. 10.3390/jcm8030400 30909477 PMC6462959

[brb33431-bib-0019] Moher, D. , Liberati, A. , Tetzlaff, J. , Altman, D. G. , & PRISMA Group . (2009). Preferred reporting items for systematic reviews and meta‐analyses: The PRISMA statement. PLoS Medicine, 6, e1000097. 10.1371/journal.pmed.1000097 19621072 PMC2707599

[brb33431-bib-0020] National Institute of Neurological Disorders and Stroke rt‐PA Stroke Study Group . (1995). Tissue plasminogen activator for acute ischemic stroke. New England Journal of Medicine, 333(24), 1581–1588. 10.1056/NEJM199512143332401 7477192

[brb33431-bib-0021] Powers, W. J. , Rabinstein, A. A. , Ackerson, T. , Adeoye, O. M. , Bambakidis, N. C. , Becker, K. , Biller, J. , Brown, M. , Demaerschalk, B. M. , Hoh, B. , & Jauch, E. C. (2019). Guidelines for the early management of patients with acute ischemic stroke: 2019 update to the 2018 guidelines for the early management of acute ischemic stroke: A guideline for healthcare professionals from the American Heart Association/American Stroke Association. Stroke; A Journal of Cerebral Circulation, 50(12), e344–e418. 10.1161/STR.0000000000000211 31662037

[brb33431-bib-0022] Powers, W. J. , Rabinstein, A. A. , Ackerson, T. , Adeoye, O. M. , Bambakidis, N. C. , Becker, K. , Biller, J. , Brown, M. , Demaerschalk, B. M. , Hoh, B. , Jauch, E. C. , Kidwell, C. S. , Leslie‐Mazwi, T. M. , Ovbiagele, B. , Scott, P. A. , Sheth, K. N. , Southerland, A. M. , Summers, D. V. , & Tirschwell, D. L. (2018). 2018 guidelines for the early management of patients with acute ischemic stroke: A guideline for healthcare professionals from the American Heart Association/American Stroke Association. Stroke; A Journal of Cerebral Circulation, 49(3), e46–e110. 10.1161/STR.0000000000000158 29367334

[brb33431-bib-0023] Quinn, T. J. , Taylor‐Rowan, M. , Coyte, A. , Clark, A. B. , Musgrave, S. D. , Metcalf, A. K. , Day, D. J. , Bachmann, M. O. , Warburton, E. A. , Potter, J. F. , & Myint, P. K. (2017). Pre‐stroke modified Rankin Scale: Evaluation of validity, prognostic accuracy, and association with treatment. Frontiers in Neurology, 8, 275. 10.3389/fneur.2017.00275 28659859 PMC5468801

[brb33431-bib-0024] Salwi, S. , Niec, J. A. , Hassan, A. E. , Lindsell, C. J. , Khatri, P. , Mocco, J. , Saver, J. L. , & Mistry, E. A. (2021). Endovascular treatment for acute stroke patients with a pre‐stroke disability: An international survey. Frontiers in Neurology, 12, 714594. 10.3389/fneur.2021.714594 34671306 PMC8520928

[brb33431-bib-0025] Saver, J. L. , Filip, B. , Hamilton, S. , Yanes, A. , Craig, S. , Cho, M. , Conwit, R. , & Starkman, S. (2010). Improving the reliability of stroke disability grading in clinical trials and clinical practice: The Rankin Focused Assessment (RFA). Stroke; A Journal of Cerebral Circulation, 41(5), 992–995. 10.1161/STROKEAHA.109.571364 PMC2930146 20360551

[brb33431-bib-0026] Stroup, D. F. , Berlin, J. A. , Morton, S. C. , Olkin, I. , Williamson, G. D. , Rennie, D. , Moher, D. , Becker, B. J. , Sipe, T. A. , & Thacker, S. B. (2000). Meta‐analysis of observational studies in epidemiology: A proposal for reporting. Meta‐analysis of observational studies in epidemiology (MOOSE) group. JAMA, 283(15), 2008–2012. 10789670 10.1001/jama.283.15.2008

[brb33431-bib-0027] The Lancet Neurology . (2020). A unified European action plan on stroke. Lancet Neurology, 19(12), 963. 10.1016/S1474-4422(20)30409-9 33181090

[brb33431-bib-0028] Tu, W. J. , Zhao, Z. , Yin, P. , Cao, L. , Zeng, J. , Chen, H. , Fan, D. , Fang, Q. , Gao, P. , Gu, Y. , Tan, G. , Han, J. , He, L. , Hu, B. , Hua, Y. , Kang, D. , Li, H. , Liu, J. , Liu, Y. , … Wang, L. (2023). Estimated burden of stroke in China in 2020. JAMA Network Open, 6(3), e231455. 10.1001/jamanetworkopen.2023.1455 36862407 PMC9982699

[brb33431-bib-0029] Turc, G. , Tsivgoulis, G. , Audebert, H. J. , Boogaarts, H. , Bhogal, P. , De Marchis, G. M. , Fonseca, A. C. , Khatri, P. , Mazighi, M. , Pérez de la Ossa, N. , & Schellinger, P. D. (2022). European Stroke Organisation—European Society for Minimally Invasive Neurological Therapy expedited recommendation on indication for intravenous thrombolysis before mechanical thrombectomy in patients with acute ischaemic stroke and anterior circulation large vessel occlusion. European Stroke Journal, 7(1), I–XXVI. 10.1177/23969873221076968 PMC8921785 35300256

[brb33431-bib-0030] Tu, W. J. , & Wang, L. D. (2023). Special writing group of China stroke surveillance report. China stroke surveillance report 2021. Military Medical Research, 10(1), 33. 37468952 10.1186/s40779-023-00463-x PMC10355019

[brb33431-bib-0031] Wardlaw, J. M. , Murray, V. , Berge, E. , & Zoppo, D. G. J. (2009). Thrombolysis for acute ischaemic stroke. Cochrane Database of Systematic Reviews (Online), 2014(4), CD000213. 10.1002/14651858.CD000213.pub3 PMC4153726 25072528

[brb33431-bib-0032] Zhang, W. , Coote, S. , Frost, T. , Dewey, H. M. , & Choi, P. M. C. (2018). Acute stroke patients with mild‐to‐moderate pre‐existing disability should be considered for thrombolysis treatment. Journal of Stroke and Cerebrovascular Diseases, 27(10), 2707–2711. 10.1016/j.jstrokecerebrovasdis.2018.05.051 30037650

