# Supplementary material for: Intravenous thrombolysis in acute ischemic stroke patients with pre‐stroke disability: A systematic review and meta‐analysis
Source: Brain Behav. 2024 Feb 15;14(2):e3431. doi: 10.1002/brb3.3431 (PMC10869883; doi:10.1002/brb3.3431)

**
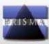
** supplementary materials 1

**PRISMA 2020 Checklist**

| **Section and Topic** | **Item#** | **Checklist item** | **where item**  **is reported** |  |
| --- | --- | --- | --- | --- |
| **TITLE** | | | |  |
| Title | 1 | Identify the report as a systematic review. | Page1 |  |
| **ABSTRACT** | | | |  |
| Abstract | 2 | See the PRISMA 2020 for Abstracts checklist. | Page2-3 |  |
| **INTRODUCTION** | | | |  |
| Rationale | 3 | Describe the rationale for the review in the context of existing knowledge. | Page3 |  |
| Objectives | 4 | Provide an explicit statement of the objective(s) or question(s) the review addresses. | Page3 |  |
| **METHODS** | | | |  |
| Eligibility criteria | 5 | Specify the inclusion and exclusion criteria for the review and how studies were grouped for the syntheses. | Page4 |  |
| Information sources | 6 | Specify all databases, registers, websites, organisations, reference lists and other sources searched or consulted to identify studies. Specify the date when each source was last searched or consulted. | Page4 |  |
| Search strategy | 7 | Present the full search strategies for all databases, registers and websites, including any filters and limits used. | Page4 |  |
| Selection process | 8 | Specify the methods used to decide whether a study met the inclusion criteria of the review, including how many reviewers screened each record and each report retrieved, whether they worked independently, and if applicable, details of automation tools used in the process. | Page4 |  |
| Data collection process | 9 | Specify the methods used to collect data from reports, including how many reviewers collected data from each report, whether they worked independently, any processes for obtaining or confirming data from study investigators, and if applicable, details of automation tools used in the process. | Page4 |  |
| Data items | 10a | List and define all outcomes for which data were sought. Specify whether all results that were compatible with each outcome domain in each study were sought (e.g. for all measures, time points, analyses), and if not, the methods used to decide which results to collect. | Page4-5 |  |
|  | 10b | List and define all other variables for which data were sought (e.g. participant and intervention characteristics, funding sources). Describe any assumptions made about any missing or unclear information. | Page4-5 |  |
| Study risk of bias  assessment | 11 | Specify the methods used to assess risk of bias in the included studies, including details of the tool(s) used, how many reviewers assessed each study and whether they worked independently, and if applicable, details of automation tools used in the process. | Page5 |  |
| Effect measures | 12 | Specify for each outcome the effect measure(s) (e.g. risk ratio, mean difference) used in the synthesis or presentation of results. | Page5 |  |
| Synthesis methods | 13a | Describe the processes used to decide which studies were eligible for each synthesis (e.g. tabulating the study intervention characteristics and comparing against the planned groups for each synthesis (item #5)). | Page5 |  |
|  | 13b | Describe any methods required to prepare the data for presentation or synthesis, such as handling of missing summary statistics, or data conversions. | Page5 |  |
|  | 13c | Describe any methods used to tabulate or visually display results of individual studies and syntheses. | Page5 |  |
|  | 13d | Describe any methods used to synthesize results and provide a rationale for the choice(s). If meta-analysis was performed, describe the model(s), method(s) to identify the presence and extent of statistical heterogeneity, and software package(s) used. | Page5 |  |
|  | 13e | Describe any methods used to explore possible causes of heterogeneity among study results (e.g. subgroup analysis, meta-regression). | Page5 |  |
|  | 13f | Describe any sensitivity analyses conducted to assess robustness of the synthesized results. | Page5 |  |
| Reporting bias  assessment | 14 | Describe any methods used to assess risk of bias due to missing results in a synthesis (arising from reporting biases). | Page5 |  |
| Certainty assessment | 15 | Describe any methods used to assess certainty (or confidence) in the body of evidence for an outcome. | Page5 |  |
| **RESULTS** | | | |  |
| Study selection | 16a | Describe the results of the search and selection process, from the number of records identified in the search to the number of studies included in the review, ideally using a flow diagram. | Page6 |  |
|  | 16b | Cite studies that might appear to meet the inclusion criteria, but which were excluded, and explain why they were excluded. | Page6 |  |
| Study characteristics | 17 | Cite each included study and present its characteristics. | Page6 |  |
| Risk of bias in studies | 18 | Present assessments of risk of bias for each included study. | Page6 |  |
| Results of individual  studies | 19 | For all outcomes, present, for each study: (a) summary statistics for each group (where appropriate) and (b) an effect estimate and its precision (e.g. confidence/credible interval), ideally using structured tables or plots. | Page6-7 |  |
| Results of syntheses | 20a | For each synthesis, briefly summarise the characteristics and risk of bias among contributing studies. | Page7-8 |  |
|  | 20b | Present results of all statistical syntheses conducted. If meta-analysis was done, present for each the summary estimate and its precision (e.g. confidence/credible interval) and measures of statistical heterogeneity. If comparing groups, describe the direction of the effect. | Page7-8 |  |
|  | 20c | Present results of all investigations of possible causes of heterogeneity among study results. | Page7-8 |  |
|  | 20d | Present results of all sensitivity analyses conducted to assess the robustness of the synthesized results. | Page7-8 |  |
| Reporting biases | 21 | Present assessments of risk of bias due to missing results (arising from reporting biases) for each synthesis assessed. | Page7-8 |  |
| Certainty of evidence | 22 | Present assessments of certainty (or confidence) in the body of evidence for each outcome assessed. | Page6 |  |
| **DISCUSSION** | | | |  |
| Discussion | 23a | Provide a general interpretation of the results in the context of other evidence. | Page8-10 |  |
|  | 23b | Discuss any limitations of the evidence included in the review. | Page10 |  |
|  | 23c | Discuss any limitations of the review processes used. | Page10 |  |
|  | 23d | Discuss implications of the results for practice, policy, and future research. | Page10 |  |
| **OTHER INFORMATION** | | | |  |
| Registration and  protocol | 24a | Provide registration information for the review, including register name and registration number, or state that the review was not registered. | Page3-4 |  |
|  | 24b | Indicate where the review protocol can be accessed, or state that a protocol was not prepared. | Page3-4 |  |
|  | 24c | Describe and explain any amendments to information provided at registration or in the protocol. | Page3-4 |  |
| Support | 25 | Describe sources of financial or non-financial support for the review, and the role of the funders or sponsors in the review. | Page10 |  |
| Competing interests | 26 | Declare any competing interests of review authors. | Page10 |  |
| Availability of data,  code and other  materials | 27 | Report which of the following are publicly available and where they can be found: template data collection forms; data extracted from included studies; data used for all analyses; analytic code; any other materials used in the review. | Page11 |  |

supplementary materials 2

**Complete search algorithm used in pubmed, embase and cochrane search**

***PUBMED***

#1. "Cerebrovascular Disorders"[MH]

#2. "Brain Ischemia"[MH] OR "Hypoxia-Ischemia, Brain"[MH] OR "Ischemic Attack, Transient"[MH]

#3. "Stroke"[MH] OR "Stroke, Lacunar"[MH] OR "Infarction, Posterior Cerebral Artery"[MH] OR "Brain Stem Infarctions"[MH] OR "Infarction, Middle Cerebral Artery"[MH] OR "Infarction, Anterior Cerebral Artery"[MH]

#4. Stroke[ti:ab] OR cerebr* vascul* infarct*[ti:ab] OR cerebrovasc* infarct*[ti:ab] OR cerebr* vasc* event*[ti:ab] OR cerebrovasc* event*[ti:ab] OR cva [ti:ab] OR transient ischemic attack*[ti:ab] OR tia[ti:ab]

#5. #1 OR #2 OR #3 OR #4

#6. "Thrombolytic Therapy"[MH] OR thromboly* therap*[ti:ab]

#7. "Fibrinolysis"[MH] OR fibrinoly*[ti:ab]

#8. “tPA” [ti:ab] OR “t-PA”[ti:ab] OR “rtPA”[ti:ab] OR “rt-PA” [ti:ab] OR “IV-tPA” [ti:ab] OR “IV rt-PA” [ti:ab]

#9. “alteplase” [ti:ab] OR “actilyse” [ti:ab] OR “activase” [ti:ab] OR “alteplasi” [ti:ab] OR “alteplasum”[ti:ab] OR “alteplasum”[ti:ab] OR “cathflo activase”[ti:ab] OR “GRTPA”[ti:ab] OR “SRT-PA”[ti:ab] OR “CAS Registry Number 105857-23-6”[ti:ab] OR “RN: 105857-23-6”[ti:ab]

#10. #6 OR #7 OR #8 OR #9

#11. Disabled Persons[MH] or Disabled Person[ti:ab] or Handicapped[ti:ab] or People with Disabilities[ti:ab] or Persons with Disabilities[ti:ab] or Persons with Disability[ti:ab] or Physically Handicapped[ti:ab] or Physically Disabled[ti:ab] or Disabled, Physically[ti:ab] or Physically Challenged[ti:ab] or Disability[ti:ab] or MRS[ti:ab] or Modified Rankin*[ti:ab] or Rankin*[ ti:ab] or ADL[MH] or activities of daily living[ti:ab] or disable[ti:ab] or disabled[ti:ab] or ADL[ti:ab] or BADL[ti:ab] or IADL[ti:ab]

#12. Humans [MeSH Terms]

#13. #5 and #10 and #12

#14 3571

***EMBASE***

#1. 'brain infarction'/exp OR 'brain ischemia'/exp OR 'cerebrovascular accident'/exp OR 'cerebral artery disease'/exp OR 'occlusive cerebrovascular disease'/exp

#2. 'lacunar stroke'/exp OR 'middle cerebral artery occlusion'/exp OR ('brain artery'/exp AND 'artery occlusion'/exp)

#3.'brain ischemia':ti,ab,kw OR 'cerebral ischemia':ti,ab,kw OR 'ischemic attack':ti,ab,kw OR 'transient ischemic attack':ti,ab,kw OR 'ischemic stroke':ti,ab,kw OR 'cerebral infarction':ti,ab,kw

#4. #1 OR #2 OR #3

#5. 'plasminogen activator'/exp OR 'fibrinolysis'/exp OR 'tissue plasminogen activator'/exp

#6. 'fibrinolytic agents':ti,ab,kw OR 'anti thrombin':ti,ab,kw OR 'tissue plasminogen activator':ti,ab,kw OR 'intravenous tissue plasminogen activator':ti,ab,kw OR 'IV tissue plasminogen activator':ti,ab,kw OR 'IV recombinant tissue plasminogen activator':ti,ab,kw OR 'Recombinant human tissue-type plasminogen activator':ti,ab,kw

#7. 'tPA':ti,ab,kw OR 't-PA':ti,ab,kw OR 'rtPA':ti,ab,kw OR 'rt-PA':ti,ab,kw OR 'IV-tPA':ti,ab,kw OR 'IV rt-PA':ti,ab,kw

#8. 'alteplase':ti,ab,kw OR 'actilyse':ti,ab,kw OR 'activase':ti,ab,kw OR 'alteplasi':ti,ab,kw OR 'alteplasum':ti,ab,kw OR 'alteplasum':ti,ab,kw OR 'cathflo activase':ti,ab,kw OR 'SRT-PA':ti,ab,kw OR ' UNII-1RXS4UE564':ti,ab,kw

#9. #5 OR #6 OR #7 OR #8

#10. 'Disability'/exp OR 'ADL disability':ti,ab,kw OR 'immobility':ti,ab,kw OR 'invalidity':ti,ab,kw OR ' limited mobility':ti,ab,kw OR 'neurodisability':ti,ab,kw OR 'physical disability':ti,ab,kw OR 'walking difficulty':ti,ab,kw OR 'work disability':ti,ab,kw OR 'Handicapped':ti,ab,kw OR 'ADL'/exp or 'activities of daily living':ti,ab,kw or 'disable':ti,ab,kw or 'disabled':ti,ab,kw or 'ADL':ti,ab,kw or 'BADL':ti,ab,kw or 'IADL':ti,ab,kw

#11. 'animals'/exp NOT 'humans'/exp

#12. #4 AND #9 AND #10 NOT #11 1429

***COCHRANE***

(thrombolysis[All Fields] OR tPA[All Fields] OR tissue plasminogen activator[All Fields]) AND (ischemic stroke[mesh] or ischemic stroke[All Fields]) AND (Handicapped[mesh] or Disabilities:ti,ab,kw or Persons with; Person:ti,ab,kw or Disabled:ti,ab,kw or Persons with Disabilities:ti,ab,kw or Handicapped:ti,ab,kw or Disability, Persons with:ti,ab,kw or People with Disability:ti,ab,kw or Disabled Person:ti,ab,kw or Persons with Disability:ti,ab,kw or Persons, Disabled:ti,ab,kw or People with Disabilities:ti,ab,kw or Disabilities, People with:ti,ab,kw or Physically Disabled:ti,ab,kw or Physically Handicapped:ti,ab,kw or Disabled, Physically:ti,ab,kw or Handicapped, Physically:ti,ab,kw or Physically Challenged:ti,ab,kw or ADL[mesh] or activities of daily living:ti,ab,kw or disable:ti,ab,kw or disabled:ti,ab,kw or ADL:ti,ab,kw or BADL:ti,ab,kw or IADL:ti,ab,kw or MRS[All Fields] or Modified Rankin*[All Fields] or Rankin*[All Fields]). 1090

supplementary materials 3

**Definitions of Symptomatic Intracranial Hemorrhage (sICH), favorable 24 h NIHSS change utilized by included studies**

| **Study** | **Definition** |
| --- | --- |
| Cooray C 2021 | SICH per the ECASS-II definition (any type of intracerebral hemorrhage on any posttreatment imaging after the start of IVT and increase of ≥4 NIHSS points or death within seven days); favorable 24 h NIHSS change were defined as a 24 h NIHSS improvement ≥4. |
| Caruso P 2020 | no special definition |
| Cooray C 2020 | SICH per the ECASS-II definition (any type of intracerebral hemorrhage on any posttreatment imaging after the start of IVT and increase of ≥4 NIHSS points or death within seven days); favorable 24 h NIHSS change were defined as a 24 h NIHSS improvement ≥4. |
| Goldhoorn RB 2018 | Symptomatic intracranial hemorrhage was assessed by the adverse events committee after evaluation of medical reports and imaging assessment. favorable 24 h NIHSS change were defined as a 24 h NIHSS improvement ≥4. |
| Zhang WW 2018 | The sICH was defined by the presence of parenchymatous hemorrhage type 2 on the 24-hour post-thrombolysis scan combined with neurological deterioration with greater than or equal to 4 points increase from baseline  NIHSS score. |
| Gensicke H 2016 | symptomatic ICH (sICH) according to criteria of the European Cooperative Acute Stroke Study (ECASS)-II trial |
| Karlinski M 2014 | significant neurological improvement at day 7 (defined as improvement of ≥ 4 points on the National Institutes of Health Stroke Scale [NIHSS] from baseline or achieving an NIHSS score of 0); sICH definitions according to SITS (ie,local or remote parenchymatous hemorrhage type 2 combined with NIHSS score ≥4 points or leading to death <22–36 hours). |
| Karlinski M 2013 | symptomatic intracranial haemorrhage (sICH) according to ECASS II definition; |
| Foell R B 2003 | SICH per the ECASS-II definition (any type of intracerebral hemorrhage on any posttreatment imaging after the start of IVT and increase of ≥4 NIHSS points or death within seven days); favorable 24 h NIHSS change were defined as a 24 h NIHSS improvement ≥4. |

supplementary materials 4

**Potential confounders adjusted in the included studies**

| Study name | Variables adjusted |
| --- | --- |
| Cooray C 2021 | age, gender, onset-to-treatment, smoke, systolic blood pressure, diastolic blood pressure, diabetes, TIA, and aspirin. |
| Cooray C 2020 | age, gender, onset-to-treatment, smoke, systolic blood pressure, diastolic blood pressure, diabetes, TIA, and aspirin. |
| Gumbinger C 2019 | age, stroke severity (measured with the NIH Stroke Scale), time from stroke onset, diabetes mellitus, previous stroke, atrial fibrillation, level of stroke care, and length of hospital stay. |
| Goldhoorn RB 2018 | age, time from onset to EVT, baseline NIHSS score, collateral score, IVT before EVT. |
| Gensicke H 2016 | age, National Institutes of Health Stroke Scale only. |
| Karlinski M 2014 | age, sex, Hypertension, Atrial Fibrillation, Congestive Heart Failure, Diabetes Mellitus, Previous Stroke, and Baseline NIHSS Score. |
| Karlinski M 2013 | age, sex, Hypertension, Atrial Fibrillation, Congestive Heart Failure, Diabetes Mellitus, Previous Stroke, and Baseline NIHSS Score. |
| Foell R B 2003 | age, initial NIHSS score, and the presence of hypertension, atrial fibrillation and previous stroke. |

supplementary materials 5

Quality assessment of included studies with the Newcastle–Ottawa Scale

| Study name | Selection | Comparability | Outcome | Overall score |
| --- | --- | --- | --- | --- |
| Cooray C 2021 | *** | ** | *** | 8/9 |
| Caruso P 2020 | *** | ** | ** | 7/9 |
| Cooray C 2020 | *** | ** | *** | 8/9 |
| Gumbinger C 2019 | *** | ** | ** | 7/9 |
| Goldhoorn RB 2018 | *** | ** | *** | 8/9 |
| Zhang WW 2018 | **** | ** | *** | 9/9 |
| Gensicke H 2016 | *** | ** | *** | 8/9 |
| Karlinski M 2014 | *** | ** | ** | 7/9 |
| Karlinski M 2013 | *** | ** | *** | 8/9 |
| Foell R B 2003 | ** | ** | *** | 7/9 |

supplementary materials 6

Funnel plots for outcomes after intravenous thrombolysis in acute ischemic stroke patients with prestroke disability. (a:24h NIHSS improvement > 4;b: death; c: symptomatic intracerebral haemorrhage;d:favorable function outcome.)

a
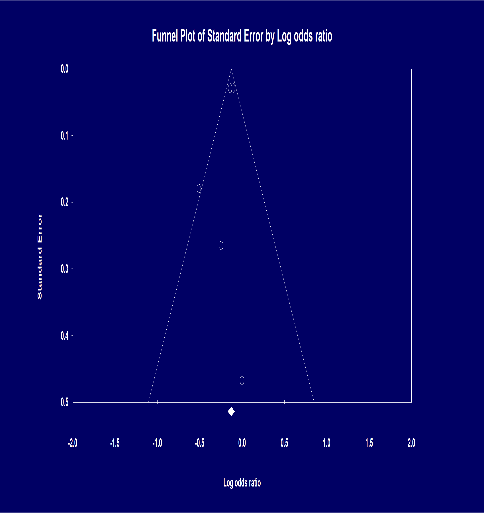
b
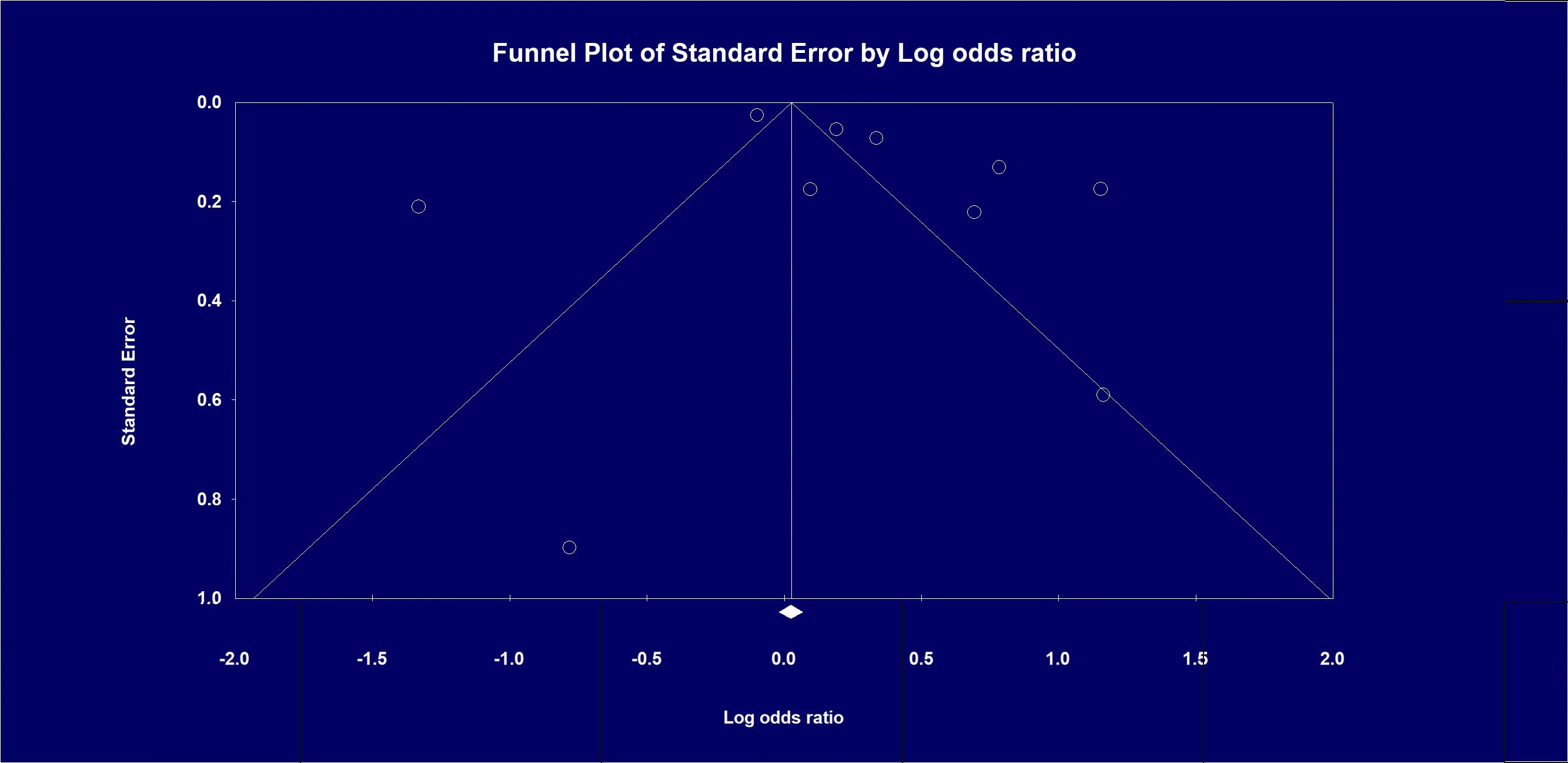
c
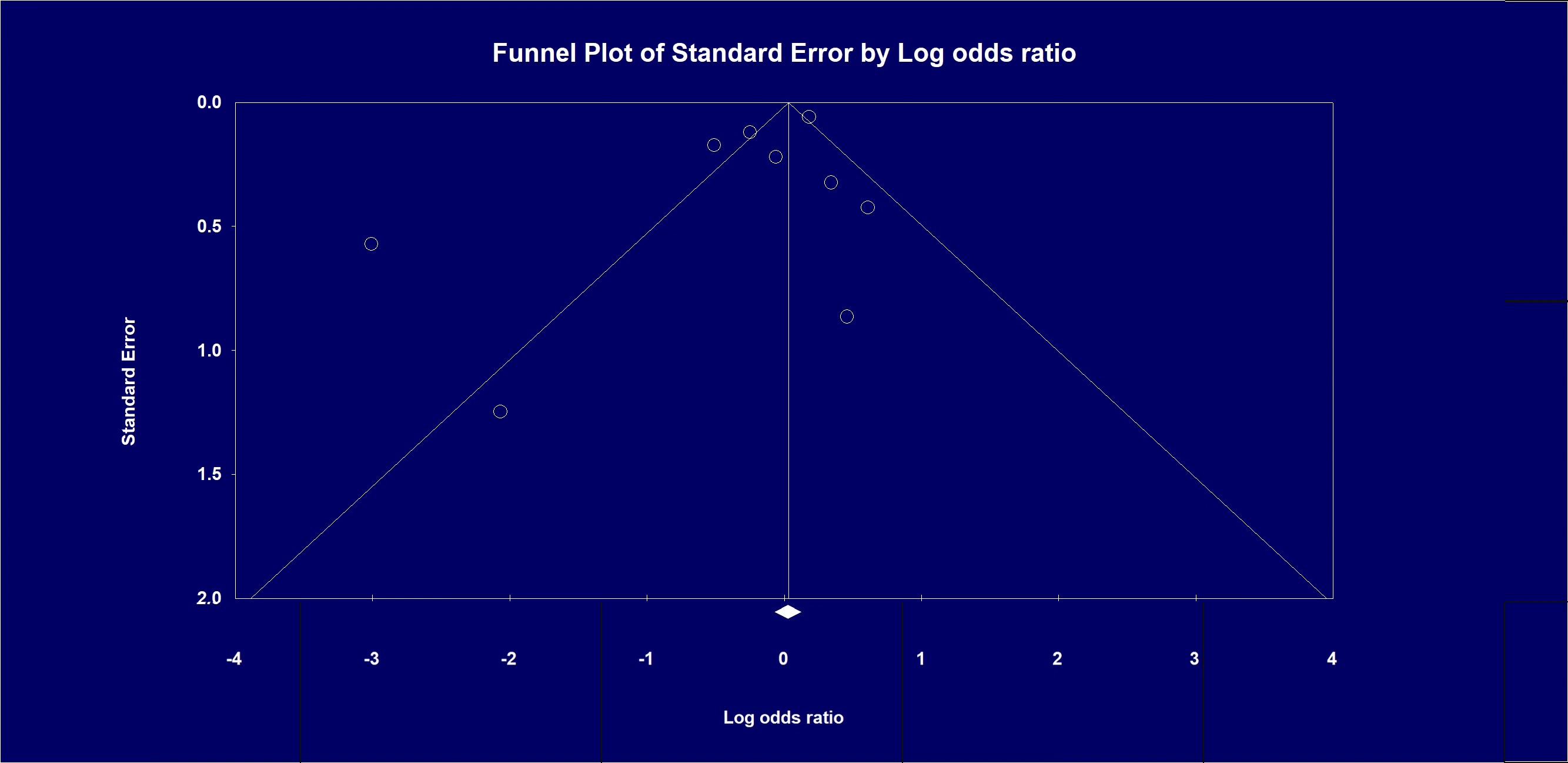
d
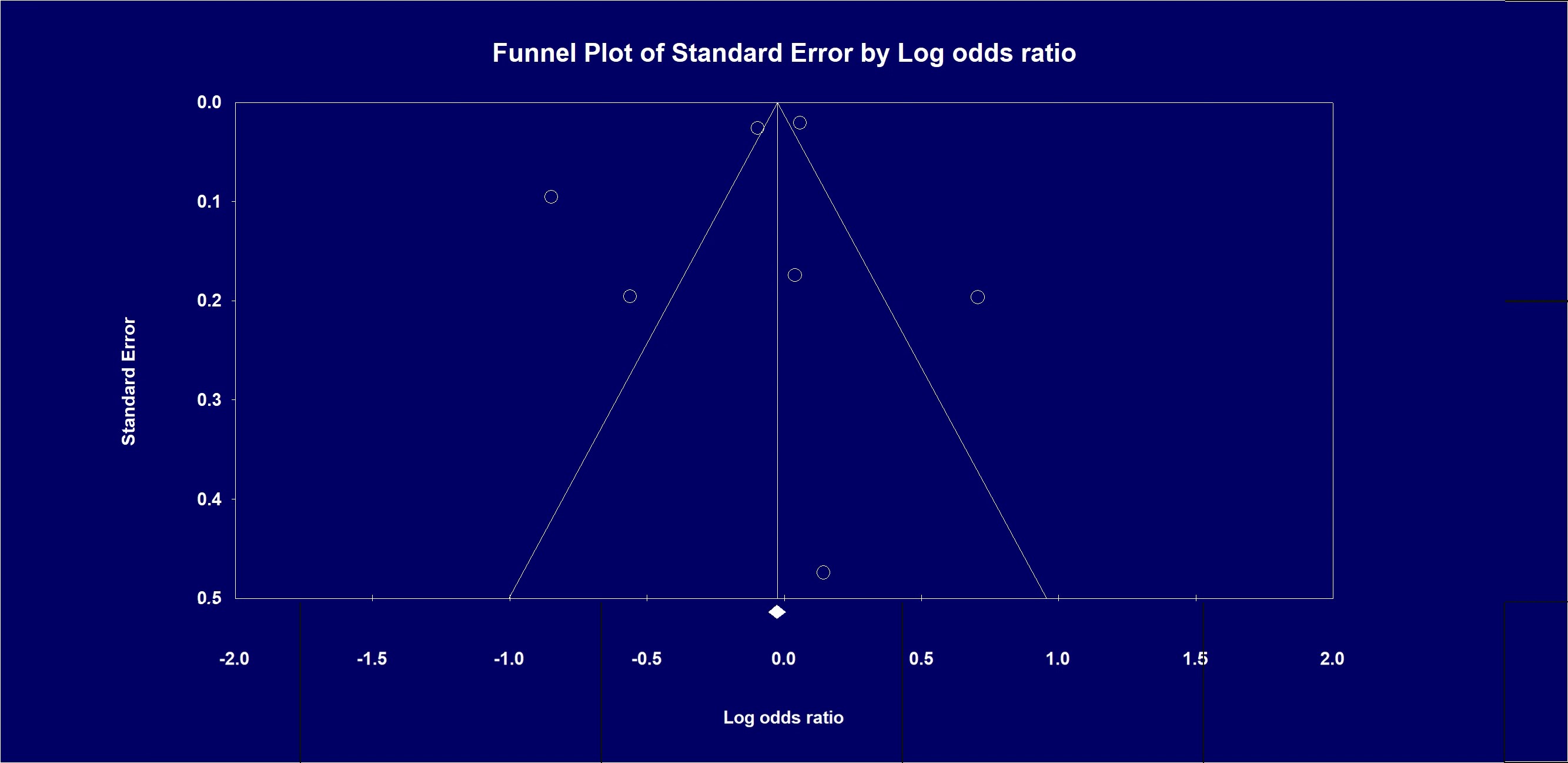


supplementary materials7

Forest Plot showing the adjusted association between prestroke disability and mortality according to time of death


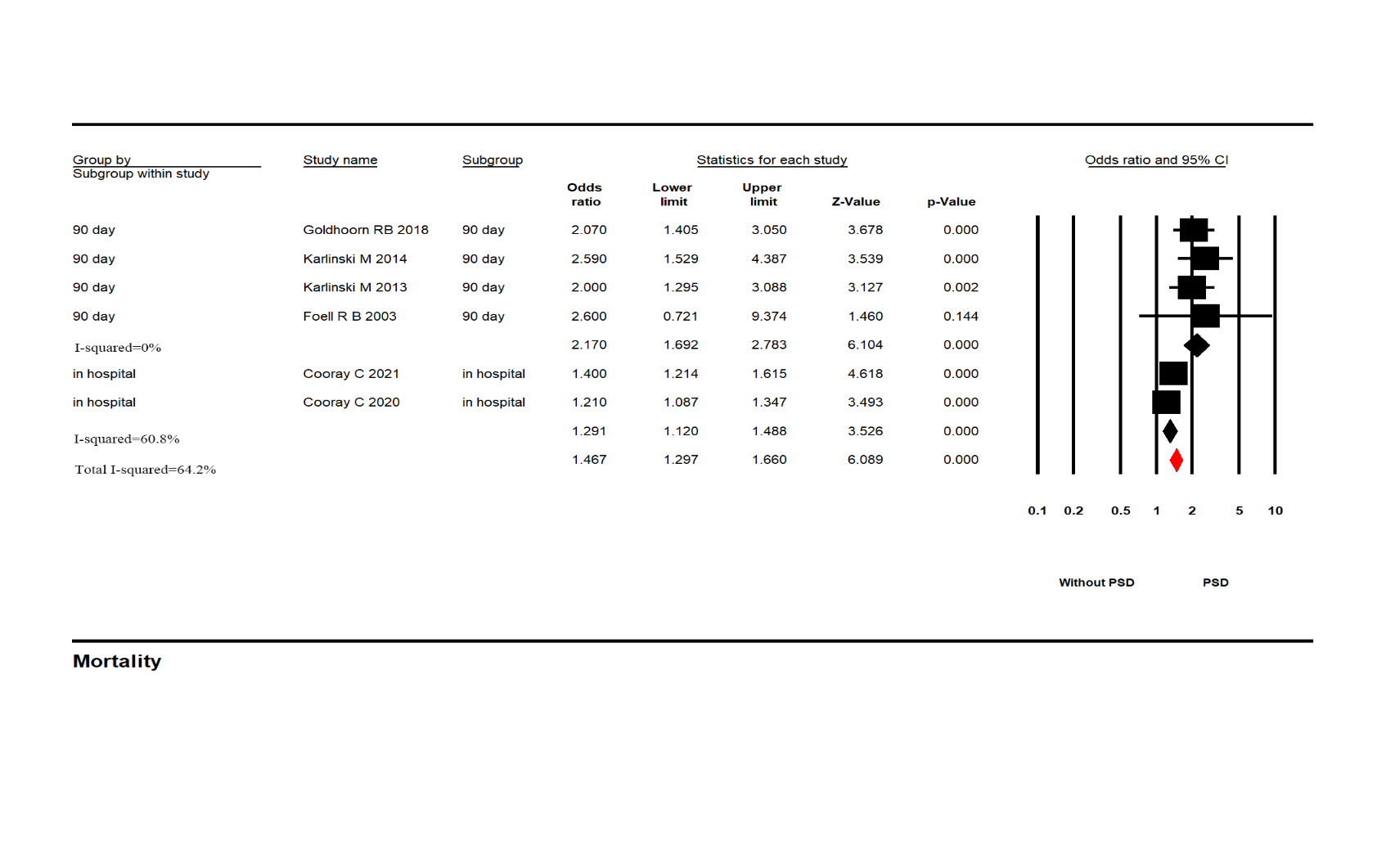


supplementary materials 8

Sensitivity analysis on the adjusted association of PSD and 24h NIHSS improvement, mortality, sICH after deduction of study.


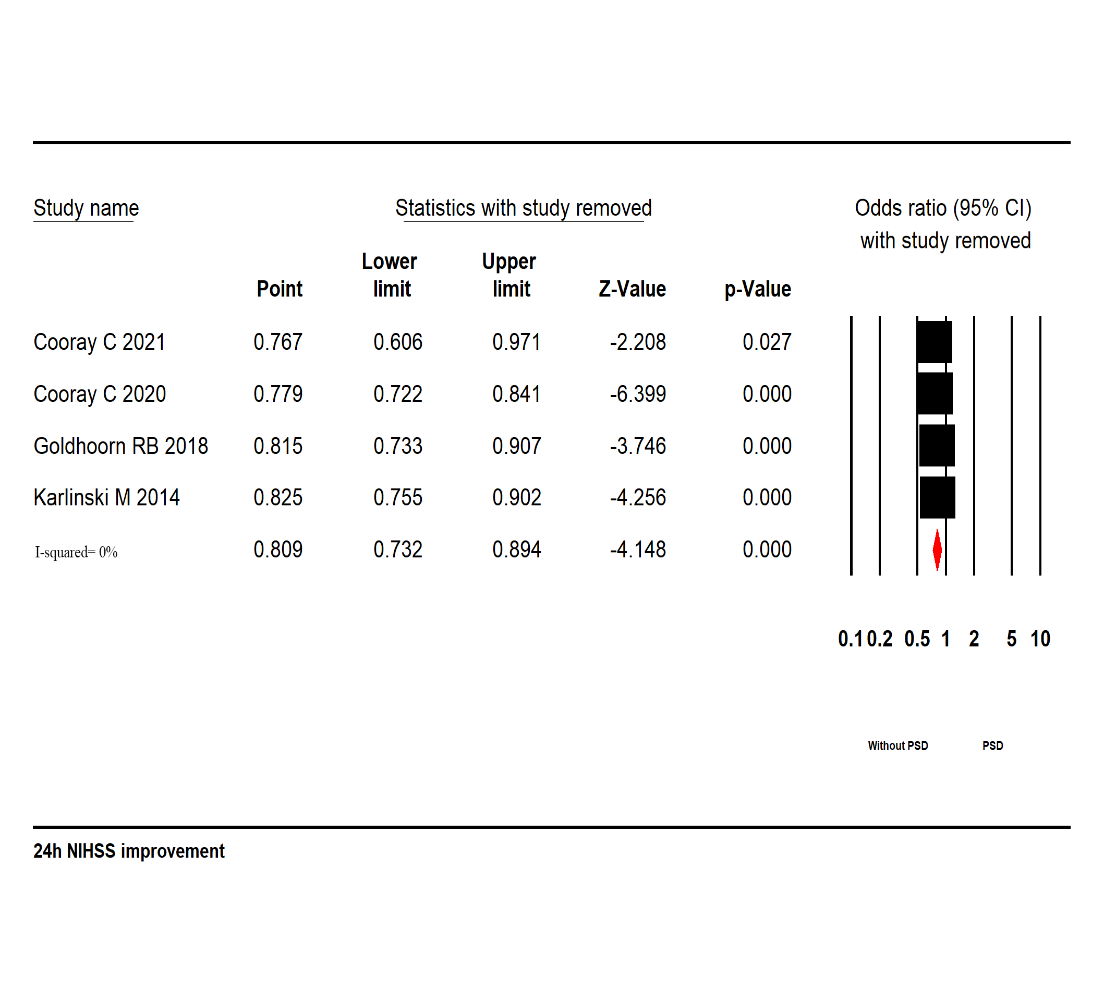

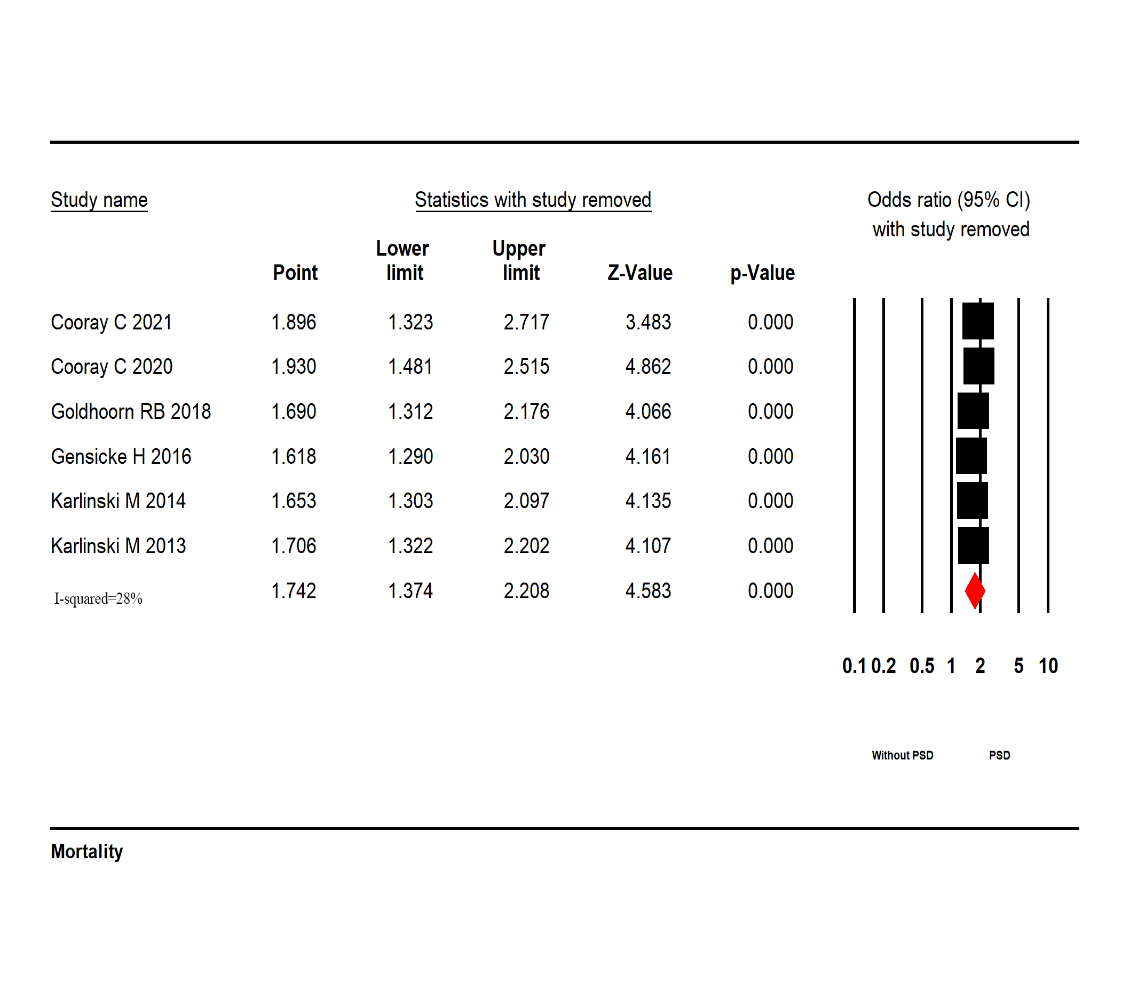


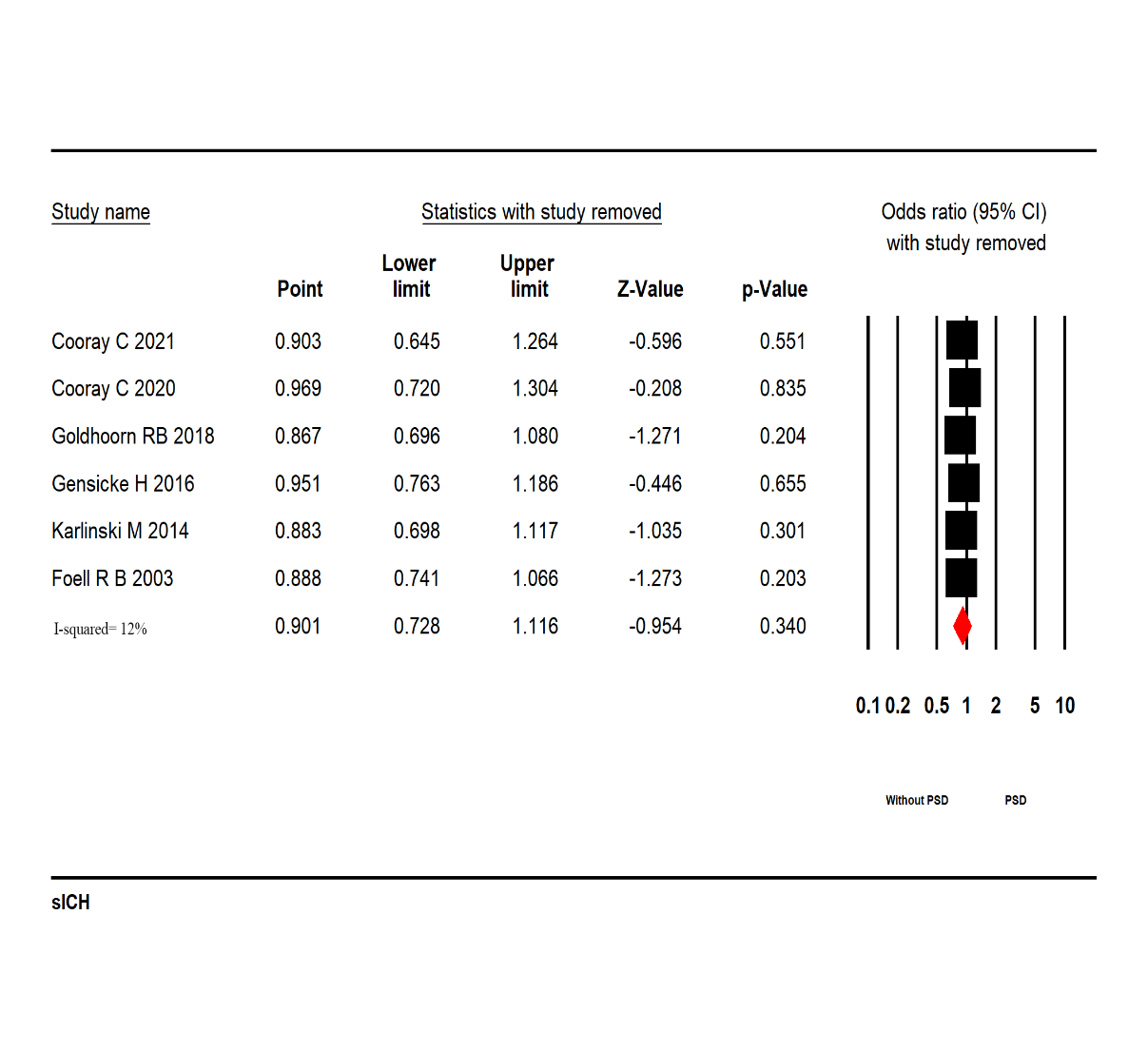


supplementary materials 9

Histogram of prognosis after endovascular therapy in acute ischemic stroke patients with disability.(Unadjusted:1.mortality; 2. FFO; 3. 24h NIHSS improvement; 4. sICH. Adjusted: 5.mortality; 6. FFO; 7. 24h NIHSS improvement; 8. sICH.)

1.
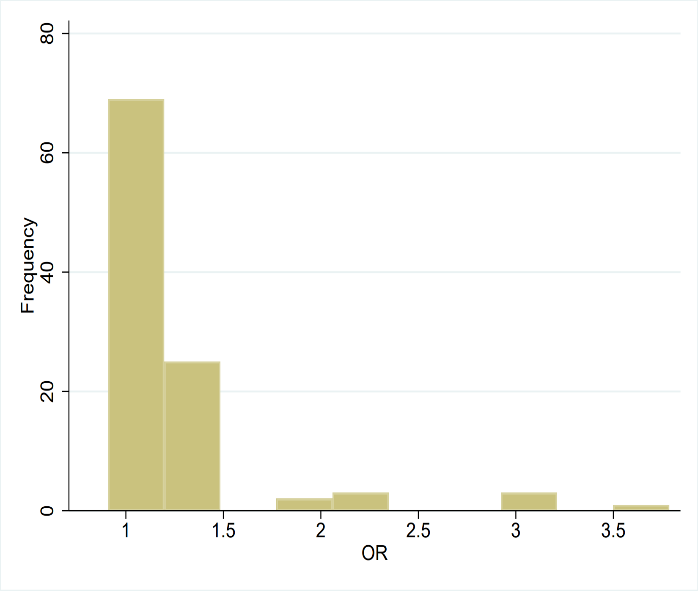
2.
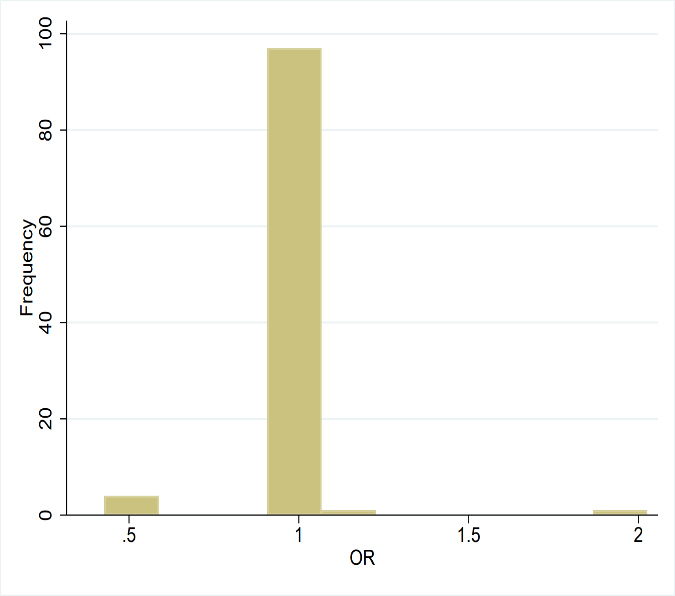


3.
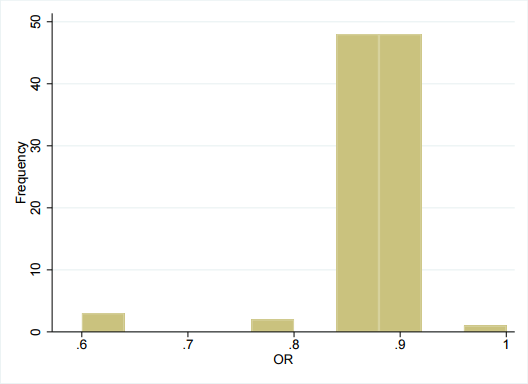
4.
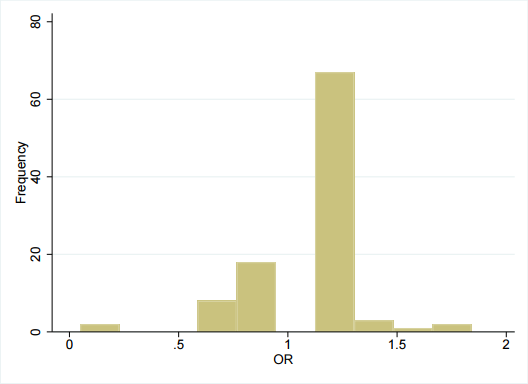


5.
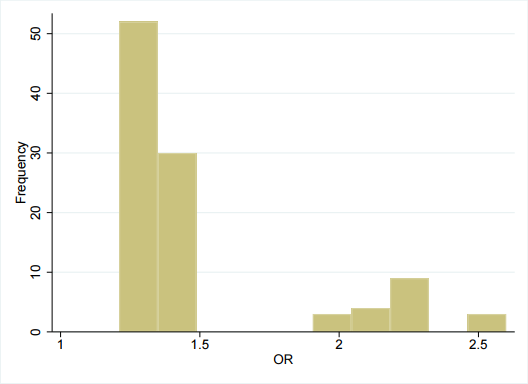
6.
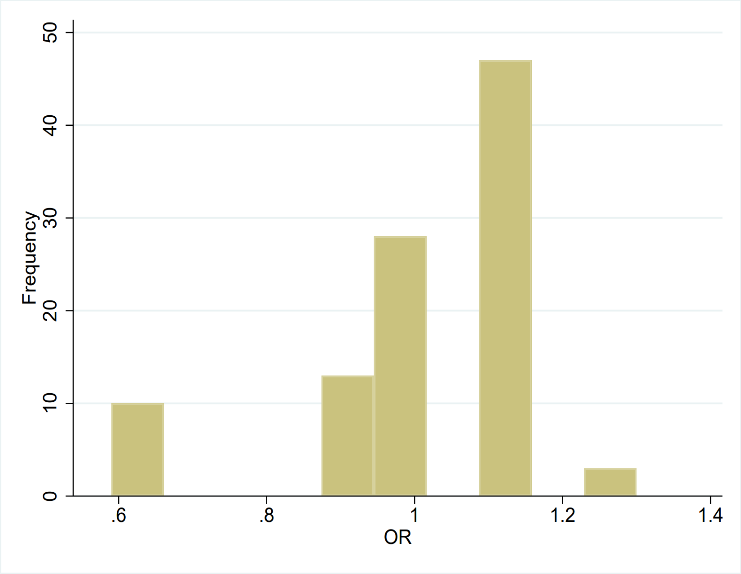


**7.** **
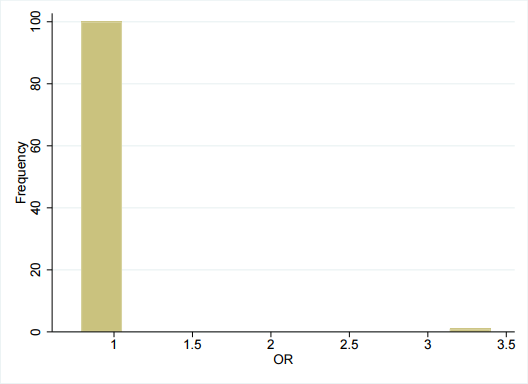
**8.
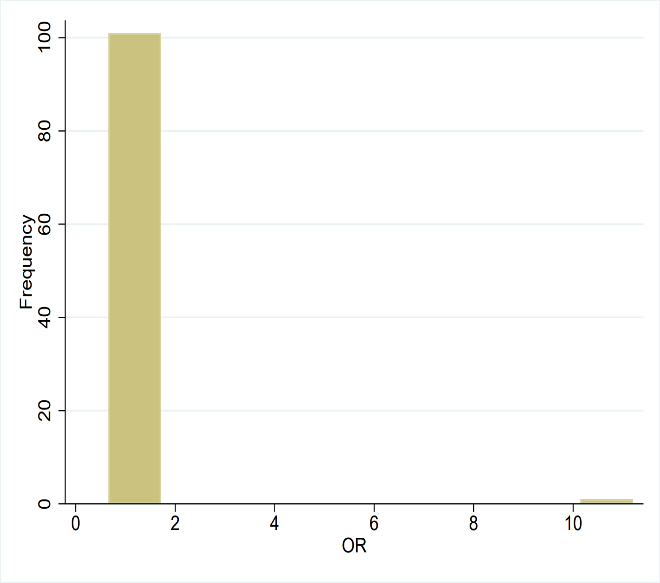

Supplement: Supplementary file 1 — Supporting Information [file BRB3-14-e3431-s001.docx]
